# Genetic Assessment of Oesophageal Safety of GLP-1 and GIP Receptor Perturbation: A Drug-Target Mendelian Randomisation Study

**DOI:** 10.3390/biomedicines14091887

**Published:** 2026-08-24

**Authors:** Hyuk Lee, Yang Won Min, Young Eun Oh, Tae-Se Kim, Byung-Hoon Min, Jun Haeng Lee, Poong-Lyul Rhee

**Affiliations:** 1 Department of Medicine, Samsung Medical Center, Sungkyunkwan University School of Medicine, Seoul 06351, Republic of Korea; 2 Samsung Precision Genome Medicine Institute, Samsung Medical Center, Seoul 06351, Republic of Korea

**Keywords:** Mendelian randomisation, incretins, glucagon-like peptide-1 receptor agonists, gastro-oesophageal reflux, Barrett’s oesophagus, oesophageal adenocarcinoma, drug safety

## Abstract

**Background/Objectives:** Glucagon-like peptide-1 (GLP-1) receptor agonists and dual glucose-dependent insulinotropic polypeptide (GIP)/GLP-1 receptor agonists are used long term for obesity and diabetes, but their oesophageal safety is uncertain: weight loss may reduce Barrett’s oesophagus and adenocarcinoma risk, whereas delayed gastric emptying may worsen reflux. We performed a genetic assessment of target-mediated oesophageal safety. **Methods:** Two-sample drug-target Mendelian randomisation used cis-expression quantitative trait loci for both receptors (31,684 participants). Outcomes were European genome-wide association studies of gastro-oesophageal reflux disease and the Barrett’s oesophagus/oesophageal adenocarcinoma axis, with a trans-ancestry squamous cell carcinoma comparator. A prespecified gated workflow required positive-control validation, and target specificity required genetic colocalisation (posterior probability of a shared causal variant ≥ 0.70). Candidate metabolic mediators were examined in two-step analyses, and findings were replicated in an independent Finnish population (FinnGen R12). **Results:** Genetically proxied GLP-1 receptor expression was not associated with reflux disease (odds ratio 0.97, 95% confidence interval 0.85–1.11), arguing against a substantial target-mediated reflux liability; the GIP receptor estimate was not estimable. For the Barrett’s oesophagus/adenocarcinoma endpoint, estimates were near the null for both receptors (GLP-1R 0.96, 0.47–1.96; GIPR 1.00, 0.42–2.37); colocalisation was uninformative because the outcome loci carried no detectable association signal, and wide intervals did not exclude moderate effects. The null reflux findings were reproduced in FinnGen. Adiposity showed the most consistent pathway association, whereas glycaemic traits did not, including when HbA1c was instrumented from a population-based genome-wide association study providing an order of magnitude more variants. **Conclusions:** GLP-1 receptor perturbation was not associated with reflux disease, providing cautious genetic reassurance. For the Barrett’s oesophagus/adenocarcinoma axis, no target-specific association was demonstrated, but colocalisation was uninformative because the outcome loci carried no detectable signal, and imprecision precludes firm safety conclusions; larger adenocarcinoma-specific studies are warranted.

## 1. Introduction

Incretin-based therapies have entered clinical practice with unusual speed and breadth. Glucagon-like peptide-1 (GLP-1) receptor agonists and dual glucose-dependent insulinotropic polypeptide (GIP)/GLP-1 receptor agonists achieve mean body-weight reductions of approximately 14–20% and are now used at population scale for obesity and type 2 diabetes [1,2,3]. As use expands among younger people, those without diabetes, and long-term or potentially lifelong treatment, the long-term oesophageal cancer safety of these agents has become a clinically important, practical question.

This uncertainty is especially pertinent to the oesophagus. Oesophageal adenocarcinoma often develops along a continuum from gastro-oesophageal reflux to Barrett’s oesophagus and then to adenocarcinoma, in which obesity and reflux are major, clinically modifiable risk factors [4]. Incretin therapies may influence this continuum in opposing directions: durable weight loss could, over years, reduce adiposity-related risk, whereas the same agents can slow gastric emptying and have been associated with increased gastro-oesophageal reflux, particularly with shorter-acting agents [5]. We therefore hypothesised that receptor perturbation may have competing oesophageal implications (reflux promotion versus adiposity reduction), with an a priori uncertain net effect.

Direct evidence cannot yet resolve this question. Trials and meta-analyses show no cancer signal [6,7], but follow-up is too short for cancers developing over decades, and observational drug studies are vulnerable to confounding by indication [8,9,10]. Drug-target Mendelian randomisation (MR) uses genetic variants near receptor genes as lifelong, naturally randomised proxies for target perturbation [11]. Fixed at conception, they largely avoid confounding and reverse causation [12]. Nonetheless, cis-variant signals may reflect linked variants rather than the intended drug target, so target-specific inference requires colocalisation. We therefore performed a genetic assessment of target-mediated oesophageal safety, asking whether lifelong, genetically proxied perturbation of GLP-1R or GIPR shows a colocalising, target-specific association with increased risk across the reflux-Barrett-adenocarcinoma continuum, and whether body weight or glycaemic traits are more consistently linked to these outcomes. Because genetic proxies do not capture drug dose, treatment duration, delayed gastric emptying, or treatment-emergent reflux, we interpret results as evidence about lifelong target perturbation, not direct estimates of the clinical effects of any specific incretin therapy.

## 2. Materials and Methods

### 2.1. Study Design and Genetic Instruments

**Figure 1:**
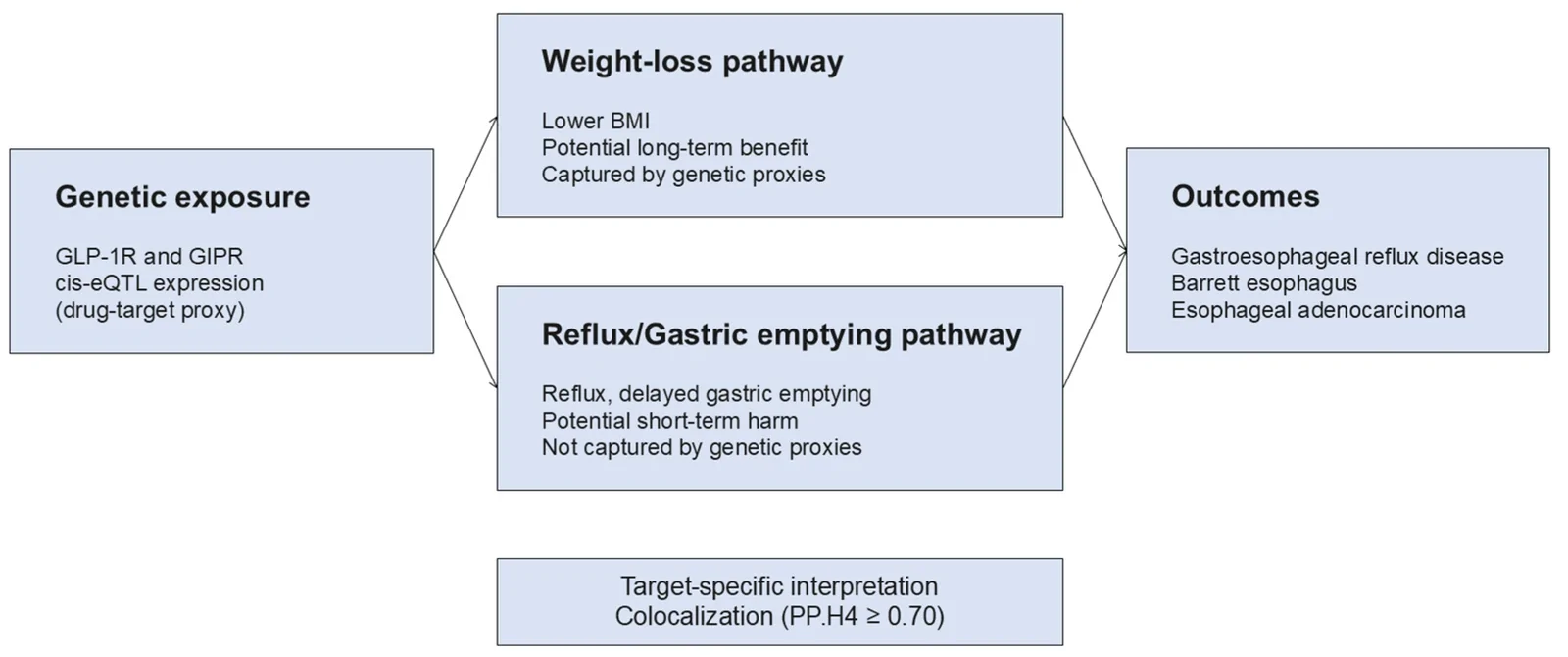
Conceptual model and interpretation framework. Genetically proxied glucagon-like peptide-1 receptor (GLP-1R) and glucose-dependent insulinotropic polypeptide receptor (GIPR) expression was evaluated as a drug-target proxy in relation to gastro-oesophageal reflux disease and the Barrett’s oesophagus/oesophageal adenocarcinoma (BO/OAC) axis. The framework distinguishes a potential long-term weight-loss pathway captured by genetic proxies from treatment-emergent reflux or delayed gastric emptying, which is not captured by germline proxies. Target-specific interpretation required colocalisation, defined as a posterior probability of a shared causal variant (PP.H4) of 0.70 or greater. OAC, oesophageal adenocarcinoma.

A two-sample, summary-level, drug-target Mendelian randomisation study was performed using a prespecified, publicly registered, gated workflow and reported in accordance with STROBE-MR [12]. The design is summarised in Figure 1 and described in detail in Supplementary Material S1. These materials include the analytic workflow, assumed causal structure, STROBE-MR checklist, and key analytic decisions (Figures S1 and S2, Tables S1 and S2). All analyses used publicly available, summary-level GWAS data. The study was prospectively registered on the Open Science Framework before any outcome data were accessed (registered 24 June 2026; https://doi.org/10.17605/OSF.IO/FRVY8). The registration specifies the instrument-selection rules, the positive-control direction gate, the colocalisation criterion, and the confirmatory versus exploratory assignment of outcomes; every departure from it is documented as a dated update on the registration and in Table S2. Oesophageal outcome data were examined only after the positive-control direction gate had been passed. The estimand was the association of genetically proxied, cis-regulated receptor expression with oesophageal outcomes, rather than the clinical effect of initiating or prescribing any specific GLP-1R or dual GIP/GLP-1R agonist.

Instruments comprised cis-expression quantitative trait loci (cis-eQTLs) for *GLP1R* and *GIPR* messenger RNA expression, derived from the eQTLGen whole-blood dataset (*n* = 31,684) [13]. Variants were selected within a 100 kb cis-window (genome build GRCh37) at genome-wide significance (*p* < 5 × 10^−8^). The primary set used independent variants (r^2^ < 0.001), whereas the sensitivity set used correlated variants (r^2^ < 0.1) with a signed linkage-disequilibrium matrix. Linkage-disequilibrium proxy substitution was not used for missing cis-eQTL instruments to avoid ambiguity in target-specific, single-instrument MR. Instruments were strong (mean F, 77.2 and 56.8; Tables S3–S6 and Figure S3). The F statistic indexes the precision of the SNP-exposure association and bounds weak-instrument bias, which acts towards the null; it does not indicate that a variant is biologically influential, and the variance in receptor expression explained was small (0.26% for *GLP1R* and 0.18% for *GIPR*). For a single-instrument Wald ratio, the test of association is determined entirely by the SNP-outcome association, so instrument strength and the scale of the exposure rescale the effect estimate but cannot generate a spurious null. Because drug-target cis-MR often relies on a single or a few cis-variants, many-instrument pleiotropy-robust estimators were not applicable. Robustness was therefore evaluated through instrument validation, direction gating, and colocalisation (Supplementary Material S1). Effect alleles were oriented to higher receptor expression; thus, estimates reflect genetically proxied higher target expression rather than pharmacologic receptor activation. Throughout, the term agonism-aligned denotes concordance with the metabolic direction expected from agonist therapy in the positive-control analyses, and nothing more. Both receptors are expressed at low levels in whole blood (GTEx v10 median transcripts per million, 0.031 for *GLP1R* and 1.012 for *GIPR*), and the blood cis-eQTL is accordingly used as a genetic proxy for cis-regulation of the receptor gene rather than as a measure of receptor function in oesophageal or gastric tissue. Both transcripts were nonetheless quantified in most of the eQTLGen sample and yielded multiple genome-wide-significant cis-eQTLs (Table S7), and tissue-specific sensitivity analyses are reported for the tissues in which the receptors are expressed (Table S8). Z scores were converted to β coefficients and standard errors from the assessed-allele frequency and per-SNP sample size (Zhu approximation); the correlated-variant analysis used a 1000 Genomes European reference panel for correlation-aware estimation, and because the targets are membrane receptors, plasma cis-protein quantitative trait loci were not used.

### 2.2. Outcomes and Metabolic Traits

The co-primary outcomes were reflux disease and a combined Barrett’s oesophagus/adenocarcinoma (BO/OAC) endpoint [14,15]. The combined BO/OAC endpoint was selected to maximise power across the Barrett’s-to-adenocarcinoma pathway, whereas OAC alone was evaluated as an underpowered sensitivity outcome [15]. Oesophageal squamous cell carcinoma (ESCC), a mechanistically distinct squamous cancer, was assessed as a descriptive comparator using a trans-ancestry dataset [16]. Candidate metabolic pathway traits included body mass index (BMI) [17], glycated hemoglobin (HbA1c) [18], and type 2 diabetes [19]. Phenotype definitions and case ascertainment followed the original GWAS publications. Variants were matched by rs identifier and harmonised to a common effect allele; intermediate-frequency palindromic variants were removed (Table 1).

### 2.3. Statistical Analysis

Before the oesophageal outcomes were examined, each instrument was tested against metabolic positive controls (type 2 diabetes, BMI, and HbA1c). The direction gate required concordance with the metabolic direction expected for incretin agonist therapy in at least 1 available positive control, with no directionally discordant estimate available; this gate was used to orient the instruments and to assess their biological plausibility, not to imply that genetically higher receptor expression is pharmacologically equivalent to receptor agonist therapy (Table S10 and Figure S4). Statistical significance was not required for every control because cis-eQTL instruments for a single receptor are few. Requiring concordance in at least one available control, without requiring statistical significance, is a permissive criterion, and the gate is therefore reported as instrument orientation rather than as formal validation. As supporting evidence, the receptor-to-HbA1c associations were also estimated with a population-based European HbA1c genome-wide association study (UK Biobank; OpenGWAS ebi-a-GCST90014006), in which both receptors were significant and agonism-aligned (GLP-1R β, −0.0746 [SE, 0.0282], *p* = 0.008; GIPR β, −0.118 [SE, 0.0341], *p* = 0.0005); the pre-registered gate itself is unchanged. A priori power was computed per outcome using the Burgess non-centrality approach (Table S9 and Figure S5) [14].

The primary MR estimate was obtained using inverse-variance weighting (IVW), which is equivalent to a Wald ratio for single-instrument analyses. To support a target-specific interpretation and reduce confounding by linked variants, colocalisation within the cis-window was required (coloc.abf), using default priors (p1 = p2 = 1 × 10^−4^; p12 = 1 × 10^−5^) [15]. The prespecified, pragmatic robustness criterion was a posterior probability of a shared causal variant (PP.H4) of 0.70 or greater; no conclusion changed at stricter thresholds (0.80 and 0.90) or when the shared-variant prior p12 was varied from 1 × 10^−6^ to 1 × 10^−4^ (Tables S11 and S12, and Figure S6). Colocalisation assumes a single causal variant per locus, and its power is bounded by outcome-locus power.

Prespecified two-step (product-of-coefficients) analyses were also performed to compare the consistency of candidate metabolic traits (BMI, HbA1c, and type 2 diabetes). Because the receptor instruments comprised a single variant or a few variants and the receptor–outcome estimates were imprecise, these estimates were considered nominal and hypothesis generating (Tables S13–S15 and Figures S7–S9). Multitissue sensitivity analyses were conducted using Genome-Tissue Expression (GTEx) tissue cis-eQTLs (Table S8 and Figure S10) [20,21]. To assess potential sample overlap between eQTLGen and the UK Biobank component of the reflux disease GWAS, we repeated the cis-MR and colocalisation in FinnGen R12, an independent Finnish population. Five analyses were added in revision: a functional coding-variant instrument set (*GLP1R* rs10305492 and *GIPR* rs1800437), reported separately from the expression-scale instruments because coding variants cannot be placed on that scale; colocalisation of each receptor cis-locus with BMI, HbA1c, and type 2 diabetes; repetition of the mediator-to-outcome and two-step analyses using a population-based European HbA1c genome-wide association study, so that the comparison with BMI is made with a substantially better-powered instrument set; completion of the registered secondary outcomes in FinnGen R12; and reporting of eQTLGen detection and testing metrics for both receptor transcripts. These were specified in a dated update to the registration before they were run and are reported irrespective of their direction (Tables S7 and S16–S20). Analyses were conducted in R version 4.5.3 (R Foundation for Statistical Computing, Vienna, Austria) using TwoSampleMR 0.7.4 [22], MendelianRandomisation 0.10.0 [23], coloc 5.2.3 [24], and ieugwasr 1.1.0 (https://mrcieu.github.io/ieugwasr/, accessed on 26 June 2026). A two-sided *p* < 0.05 was used as descriptive statistical evidence, whereas target-specific interpretation required the prespecified colocalisation criterion.

## 3. Results

### 3.1. Instrument Strength, Positive Controls, and Power

Among the available positive-control estimates, both receptor instruments passed the prespecified direction gate: glucagon-like peptide-1 receptor (GLP-1R) was associated with lower BMI (*p* = 0.002), and glucose-dependent insulinotropic polypeptide receptor (GIPR) was associated with lower HbA1c (*p* < 0.001); none of the available estimates were directionally discordant (Table S10 and Figure S4). GIPR-specific inference was more limited because estimates for GIPR-BMI, GIPR-type 2 diabetes, and GIPR-reflux disease were unavailable. Both instruments were strong (mean F-statistics were 77.2 and 56.8 for GLP-1R and GIPR, respectively; Table S3 and Figure S3) but were based on a single or a few cis-variants. A priori power was adequate for the estimable GLP-1R-reflux disease analysis. Nonetheless, the single-instrument BO/OAC analyses were underpowered (power, 0.13–0.17), and the correlated GLP-1R instrument set partly improved power for BO/OAC (Table S9 and Figure S5).

### 3.2. No Target-Specific Association for Reflux Disease or the Barrett’s/OAC Endpoint

**Figure 2:**
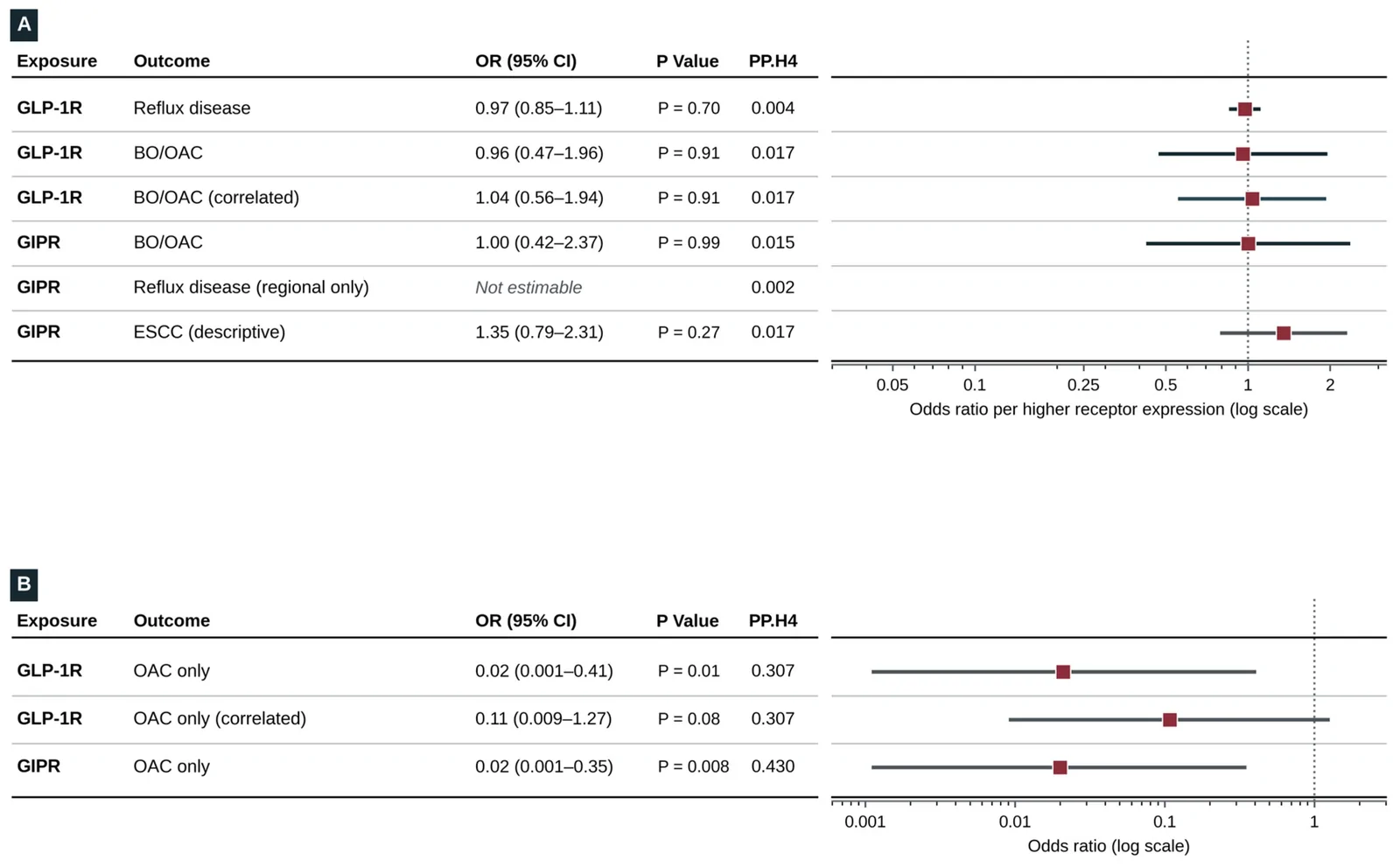
Drug-target Mendelian randomisation estimates and colocalisation. (**A**) Primary and co-primary analyses. (**B**) OAC-only sensitivity analyses, shown on an expanded log scale and not interpreted as target specific. Estimates are odds ratios (ORs) per genetically proxied higher receptor expression. Squares indicate point estimates and horizontal lines indicate 95% confidence intervals (CIs); the dotted vertical line marks the null (OR = 1). Target-specific interpretation required colocalisation, defined as PP.H4 ≥ 0.70. GIPR-reflux disease was not estimable; its PP.H4 value is shown only as a descriptive regional colocalisation result. BO/OAC, Barrett’s oesophagus/oesophageal adenocarcinoma; ESCC, oesophageal squamous cell carcinoma; GIPR, glucose-dependent insulinotropic polypeptide receptor; GLP-1R, glucagon-like peptide-1 receptor; OAC, oesophageal adenocarcinoma; PP.H4, posterior probability of a shared causal variant.

Genetically proxied GLP-1R expression was not associated with reflux disease (odds ratio [OR], 0.97 [95% confidence interval [CI], 0.85–1.11]). GIPR-reflux disease could not be estimated because the *GIPR* cis-eQTL instrument was unavailable in the reflux disease GWAS after harmonisation. For the combined BO/OAC endpoint, estimates for both receptors were close to the null (GLP-1R: OR, 0.96 [95% CI, 0.47–1.96]; GIPR: OR, 1.00 [95% CI, 0.42–2.37]). No receptor–outcome pair met the prespecified colocalisation threshold (PP.H4 ≥ 0.70): PP.H4 was 0.004 for GLP-1R-reflux disease, 0.017 for GLP-1R-BO/OAC, and 0.015 for GIPR-BO/OAC (Table 2 and Table S11, Figure 2). These outcome loci, however, carried no detectable association signal (maximum −log10 *p*, 1.2 to 2.7, with no variant reaching *p* < 1 × 10^−5^), and posterior mass concentrated on association with the eQTL alone (PP.H1, 0.95 to 0.99); the colocalisation analysis was therefore uninformative for these endpoints rather than negative (Table S17). The GIPR-reflux disease locus was the exception: it carried a detectable outcome signal (minimum *p* = 3.6 × 10^−7^, four variants at *p* < 1 × 10^−5^), and there, colocalisation was informative and indicated distinct causal variants (PP.H3, 0.79). The correlated-instrument estimate for GLP-1R on BO/OAC was concordant (OR, 1.04 [95% CI, 0.56–1.94]).

In an independent Finnish population (FinnGen R12), which does not overlap the UK Biobank component of the reflux disease GWAS, genetically proxied GLP-1R and GIPR perturbation again showed null associations with reflux disease (odds ratios 0.89–0.99) and no outcome met the prespecified colocalisation threshold (PP.H4 < 0.13), reproducing the primary findings (Table 3).

The OAC-only estimates were extreme and nominally protective, yet they were not considered target specific. These estimates relied on single-variant Wald ratios from a dataset with only 240 OAC cases (Table 1). The estimated effects had implausibly large magnitudes for expression proxies, were not reproduced using correlated-instrument estimation (OR, 0.11, 95% CI, 0.009–1.27, *p* = 0.08), and did not meet the prespecified colocalisation criterion. Under the default prior, PP.H4 remained below the threshold for the OAC-only estimates (GLP-1R, 0.307; GIPR, 0.430). PP.H4 rose above 0.70 only under a permissive shared-variant prior; nevertheless, these single-variant, underpowered sensitivity estimates were not interpreted as target specific (Tables S11 and S12).

### 3.3. Functional Coding-Variant Instruments

Two established functional coding variants were evaluated as a separate instrument set: *GLP1R* rs10305492 (p.Ala316Thr) and *GIPR* rs1800437 (p.Glu354Gln). Because these are missense rather than cis-eQTL variants, they cannot be placed on the cis-regulated expression scale of the primary instrument; they are therefore reported as a distinct sensitivity set, with per-allele associations reported directly and the strength of the available glycaemic anchor stated (Table S16).

For GLP-1R, the HbA1c-lowering A allele of rs10305492, which is the agonism-aligned direction, was not associated with reflux disease (β, −0.009; SE, 0.019; *p* = 0.63), the combined BO/OAC endpoint (β, −0.028; SE, 0.102; *p* = 0.78), or adenocarcinoma alone (β, −0.243; SE, 0.417; *p* = 0.56). Scaled ratio estimates are not reported for either variant, because the available glycaemic anchor is weak (rs10305492, anchor F = 7.2; rs1800437, F < 1), so the per-allele associations are the basis for inference (Table S16). The functional variant therefore reproduced the null obtained with the expression-based instrument.

For GIPR, rs1800437 was only weakly associated with HbA1c in the anchor dataset (*p* = 0.71; anchor F < 1), so a scaled ratio estimate was not interpretable and is not reported. Per-allele associations were null for every cancer endpoint (BO/OAC, *p* = 0.58; adenocarcinoma alone, *p* = 0.98; ESCC, *p* = 0.94). The reduced-function Gln354 (C) allele was, however, associated with lower reflux risk (β, −0.031; SE, 0.006; *p* = 3.6 × 10^−7^). This estimate is quantitatively consistent with mediation through adiposity: the same allele lowers BMI by 0.033 SD (*p* = 7.7 × 10^−51^), which with the BMI-reflux disease estimate (0.758) predicts β = −0.025. It also illustrates that reduced GIPR function and higher *GIPR* expression can both align with a metabolically favourable profile, so the direction of GIPR perturbation is not straightforward.

### 3.4. Colocalisation of the Receptor Cis-Loci with Metabolic Traits

To test whether the cis-eQTL instruments tag receptor-relevant biology rather than linked variants, which is a particular concern at the *GIPR* locus because it lies adjacent to QPCTL, colocalisation was repeated against BMI, HbA1c, and type 2 diabetes (Table S18).

The *GIPR* cis-eQTL colocalised with type 2 diabetes (PP.H4 = 0.99), indicating a shared causal variant and supporting the instrument as a proxy for receptor biology; this also supplies the GIPR-type 2 diabetes validation that was unavailable as an MR estimate because the instrument variant was absent from that GWAS after harmonisation. By contrast, the BMI signal at the same locus arose from a distinct causal variant (PP.H3 = 1.00), so BMI-related association in the *GIPR*-QPCTL region should not be attributed to *GIPR* expression; accordingly, no GIPR-based BMI pathway was estimated. HbA1c likewise favoured distinct variants (PP.H3 = 0.98); the window contains 42 genome-wide-significant HbA1c variants, however, which violates the single-causal-variant assumption of coloc.abf and favours PP.H3, so multi-signal colocalisation would be required to resolve this. At the *GLP1R* locus, no metabolic trait reached genome-wide significance (maximum −log10 *p*, 3.8 to 6.5), so colocalisation could not adjudicate there.

### 3.5. Adiposity Was the Most Consistent Metabolic Pathway Signal

**Figure 3:**
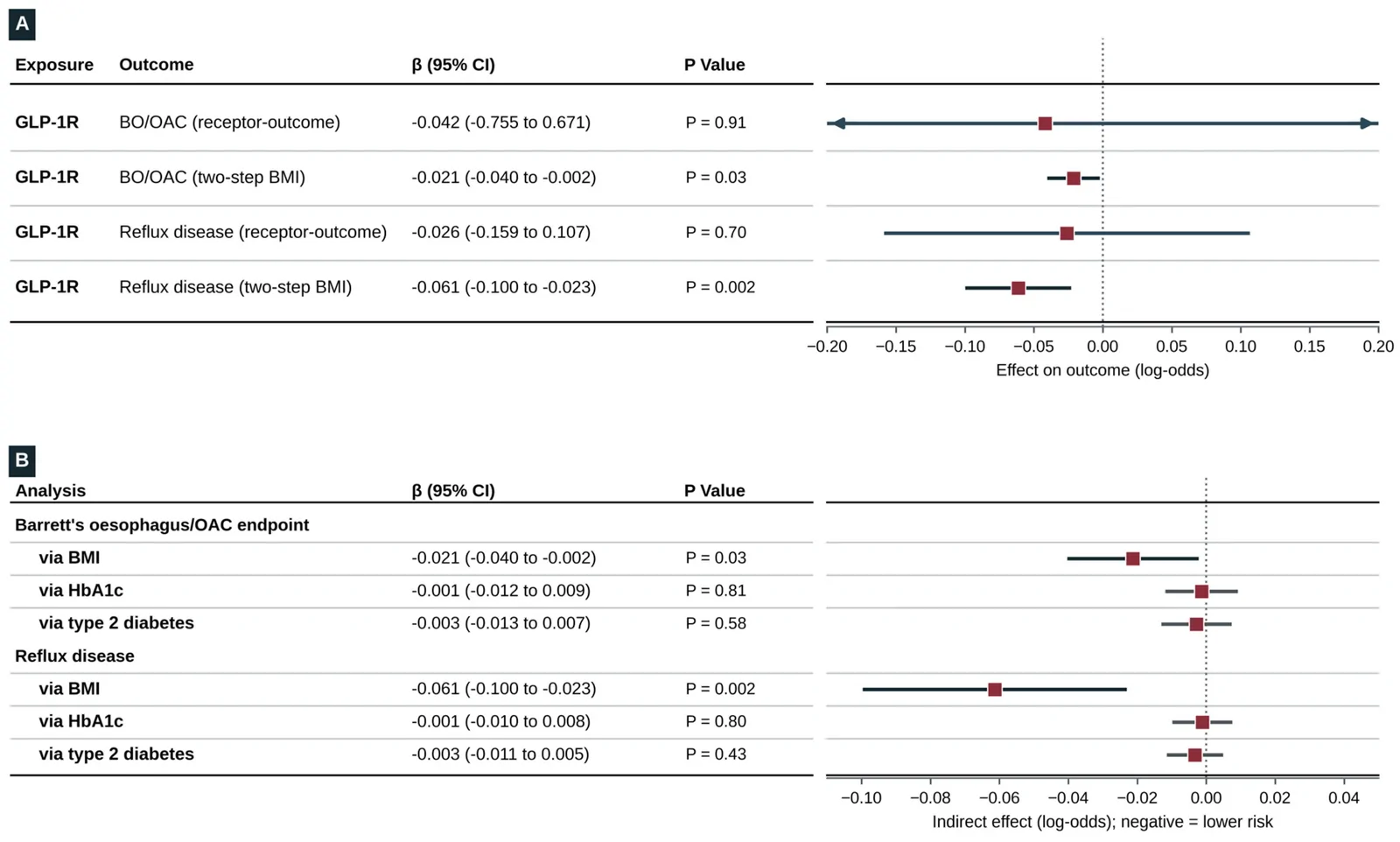
Exploratory metabolic pathway analysis. (**A**) Receptor–outcome estimates compared with GLP-1R-based two-step BMI pathway estimates for BO/OAC and reflux disease. (**B**) GLP-1R-based two-step indirect estimates by candidate metabolic trait (receptor-to-trait-to-outcome estimates, not the trait-to-outcome estimates in Table S14). These estimates assess pathway consistency and should not be interpreted as mediation of a validated receptor effect, because receptor–outcome associations did not meet the colocalisation criterion; arrows denote intervals beyond the displayed axis. Squares indicate point estimates and horizontal lines indicate 95% CIs; beta is on the log-odds scale, and negative values indicate lower risk. BMI, body mass index; BO/OAC, Barrett’s oesophagus/oesophageal adenocarcinoma; CI, confidence interval; GLP-1R, glucagon-like peptide-1 receptor; HbA1c, glycated haemoglobin.

Because no receptor–outcome association met the colocalisation criterion, and because colocalisation was uninformative at these loci, pathway estimates were used only to compare the consistency of candidate metabolic traits across oesophageal outcomes, rather than to establish mediation of a validated receptor effect. Higher genetically predicted BMI was strongly associated with reflux disease (log-odds β, 0.76, *p* < 0.001) and was associated with BO/OAC in the primary IVW analysis (log-odds β, 0.26, *p* = 0.002), whereas HbA1c and type 2 diabetes were not associated with BO/OAC. Consistent with the GLP-1R-BMI positive-control association, the GLP-1R → BMI → outcome pathway yielded nominally protective two-step estimates for BO/OAC (indirect estimate, −0.021, *p* = 0.03) and reflux disease (indirect estimate, −0.061, *p* = 0.002) (Figure 3). GIPR-specific pathway estimation was not possible because GIPR-BMI estimates were unavailable. Sensitivity analyses supported the BMI-reflux disease association in weighted median and MR-Egger analyses, whereas the BMI-BO/OAC association was weaker and not confirmed (Table S15): it rested on the inverse-variance weighted estimate alone, was not corroborated by weighted median (β, 0.083; *p* = 0.59) or MR-Egger (β, 0.166; *p* = 0.47), and showed significant heterogeneity (Cochran Q, *p* = 0.004) and MR-PRESSO global test (*p* = 0.003), so it is not interpreted as robust. Because the BMI-mediated indirect estimate for reflux disease exceeded the total effect, the implied direct effect was positive across path-b estimators (0.014 to 0.035), but its confidence interval included the null throughout (inverse-variance weighted estimate, 0.035; 95% CI, −0.103 to 0.173; *p* = 0.62) and it is reported as hypothesis generating (Table S19). The MR-Egger intercept for BMI-reflux disease indicates directional pleiotropy (0.0047, *p* = 1.6 × 10^−5^), so the MR-Egger-based indirect estimate is treated as the pleiotropy-robust bound of that range. Among the metabolic traits evaluated, adiposity showed the most consistent association pattern, with stronger support for reflux disease than for the BO/OAC endpoint. This contrast persisted when HbA1c was instrumented from a population-based European genome-wide association study, which provided an order of magnitude more variants (109 and 333 for reflux disease and BO/OAC, respectively, compared with 9 and 29 in the within-sibship dataset, although still fewer than the 481 and 485 available for BMI): HbA1c remained unassociated with both outcomes, and the two-step estimates through HbA1c were null (Table S19).

### 3.6. Descriptive Comparator and Tissue Sensitivity

The GIPR-ESCC descriptive comparator showed no signal (OR 1.35; 95% CI 0.79–2.31; Figure S9). However, this analysis was underpowered, trans-ancestry, and was not interpreted causally. In tissue-specific analyses, a pancreatic GLP-1R instrument yielded a BO/OAC estimate concordant with the blood-based analysis (OR, 1.02, 95% CI, 0.86–1.21, Table S8 and Figure S10), whereas no usable *GIPR* tissue cis-eQTL was detected. These analyses offer limited consistency checks but do not resolve tissue-specific pharmacology. In addition, blood eQTLs may not fully represent oesophageal or gut biology.

### 3.7. Pre-Registered Secondary Outcomes

Three registered secondary outcomes, gastric cancer, peptic ulcer disease, and chronic gastritis, had a priori power below 0.17 at the pre-analysis power assessment and were designated exploratory. They were not analysed for the original report and were completed during revision in FinnGen R12, ancestry-matched to the European instruments (Table S20). No association reached nominal significance for either receptor: gastroduodenal ulcer (GLP-1R: OR, 1.04 [95% CI, 0.73–1.46]; GIPR: OR, 1.46 [0.96–2.22]), chronic gastritis (GLP-1R: OR, 0.87 [0.62–1.22]; GIPR: OR, 1.03 [0.68–1.56]), and gastric cancer (GLP-1R: OR, 1.47 [0.68–3.20]; GIPR: OR, 0.76 [0.29–1.94]). As for the oesophageal outcomes, the regional outcome signal was undetectable (maximum −log10 *p*, 2.2 to 3.4) and colocalisation was uninformative (PP.H4, 0.007 to 0.032). Gastroparesis and upper-gastrointestinal dysmotility could not be analysed because no adequately powered GWAS exists.

## 4. Discussion

This study was designed as a genetic assessment of target-mediated oesophageal safety, and its conclusions differ by outcome according to precision. For gastro-oesophageal reflux disease, the best-powered outcome, genetically proxied GLP-1R perturbation showed a null association with a reasonably precise confidence interval (odds ratio 0.97, 95% CI 0.85–1.11), arguing against a substantial target-mediated reflux liability. For the Barrett’s oesophagus/oesophageal adenocarcinoma axis, no target-specific association was demonstrated for either receptor; the confidence intervals were wide and colocalisation was uninformative because the outcome loci carried no detectable signal, so these estimates indicate an absence of evidence rather than evidence excluding moderate effects. The adenocarcinoma-only analysis was underpowered and is regarded as exploratory: its extreme, nominally protective estimates did not meet the colocalisation criterion. Across outcomes, adiposity showed the most consistent metabolic association whereas glycaemic traits did not, a contrast that persisted when HbA1c was instrumented from a substantially better-powered population-based dataset (Table S19), suggesting any long-term oesophageal benefit of incretin therapy, if present, would be expected to track durable weight loss.

**Figure 4:**
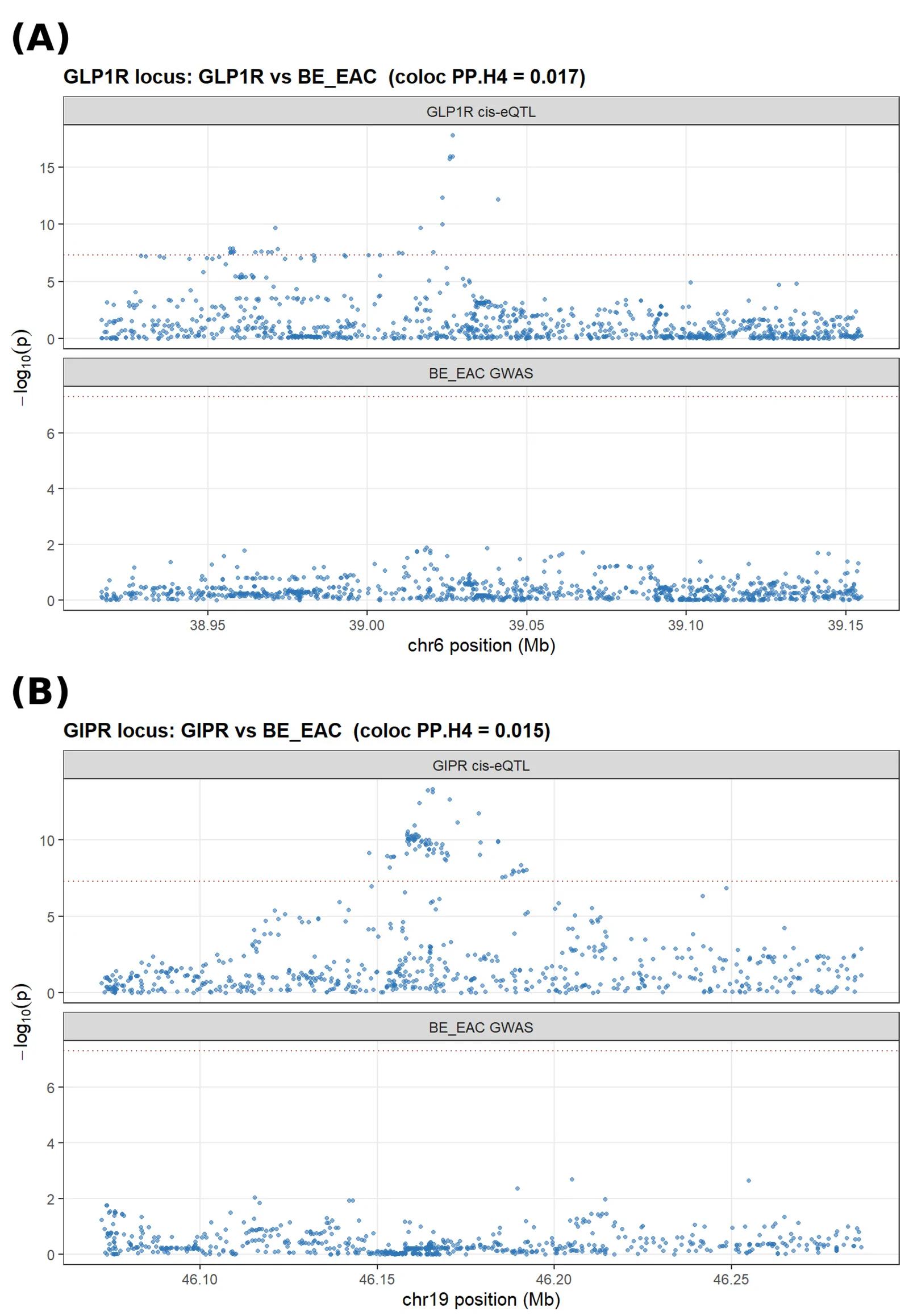
Cis-locus colocalisation plots for receptor expression and the Barrett’s oesophagus/oesophageal adenocarcinoma (BO/OAC) endpoint. (**A**) *GLP1R* cis-locus: the upper panel shows the *GLP1R* cis-expression quantitative trait locus (cis-eQTL) association and the lower panel the corresponding BO/OAC genome-wide association study association across the cis-window (*x*-axis, chromosomal position; *y*-axis, −log10 *p*); the posterior probability of a shared causal variant was low (PP.H4 = 0.017), but the BO/OAC panel shows no detectable association across the window, so colocalisation is uninformative rather than evidence against a shared causal variant. (**B**) *GIPR* cis-locus: the corresponding *GIPR* cis-eQTL and BO/OAC associations; PP.H4 was likewise low (0.015), and the same limitation applies. The label BE_EAC in the panels denotes the BO/OAC dataset.

The genetic evidence bears on the safety question in two ways. We found no target-specific evidence of increased Barrett’s/OAC risk for either receptor (Figure 4), although colocalisation could not adjudicate target specificity at these loci, and genetically proxied GLP-1R perturbation was not associated with reflux disease (the GIPR-reflux disease MR estimate was not estimable), so we do not infer a causal receptor effect from the receptor–outcome estimates. Pathway analysis does not make these estimates causal; it evaluates which metabolic trait is most consistently linked to these outcomes using larger instrument sets for BMI and glycaemic traits. Genetically predicted higher body weight was robustly associated with reflux disease and, more weakly, with Barrett’s/adenocarcinoma, whereas glucose traits were not. Because these drugs lower body weight, the protective associations reported observationally, insofar as they reflect biology rather than residual bias, may be more compatible with weight loss than with glucose lowering. The contrast between the imprecise, single-variant receptor estimates and the weight-linked signal, which drew on many BMI instruments, is expected; the most cautious reading is no target-specific risk signal but a hypothesis of an adiposity-linked pathway. One feature of the two-step estimates is worth noting: the BMI-mediated indirect effect on reflux disease exceeded the imprecise total effect, implying a small positive direct effect (0.014 to 0.035 across path-b estimators). Its interval includes the null throughout, so it is hypothesis generating only, but it is compatible with a non-adiposity pathway offsetting part of the weight-mediated protection, which is the competing-mechanism possibility set out in the Introduction.

The genetic findings are directionally consistent with some clinical and epidemiologic studies, although these differ in design, outcome definitions, comparator groups, and susceptibility to bias (Table S21; illustrative rather than systematic). Randomised trials and meta-analyses have not shown an excess malignancy signal with GLP-1 receptor agonists [6,7], whereas pharmacoepidemiologic findings for oesophageal cancer are mixed, including a Nordic case–control analysis that reported divergent associations for adenocarcinoma and squamous cell carcinoma [8,9,10], and other cohorts reported lower incidence (hazard ratios of 0.74 and 0.60) [25,26]. Broader cohorts of obesity-associated and gastrointestinal cancers have reported similar null-to-protective associations, although outcomes and comparators differ across studies [27,28,29]. The role of obesity in Barrett progression and adenocarcinoma is well established [4], and bariatric-surgery data are consistent with lower adenocarcinoma incidence after substantial weight loss [30]. This comparison across study designs assesses consistency, not proof; our weight-linked pathway offers a mechanism (adiposity rather than glucose) consistent with these observations [31].

The reflux liability of incretin therapy should be considered separately from cancer risk. Treatment-emergent reflux is an acute, dose- and drug-dependent clinical problem related to delayed gastric emptying; it is most pronounced with shorter-acting agents [5]. This pattern aligns with pharmacovigilance analyses reporting gastrointestinal adverse-event signals for these agents (Figure S11) [32]. Germline variation does not model this symptom pathway. The absence of a genetic target-specific cancer signal should not prompt clinicians to dismiss new or worsening reflux, regurgitation, dysphagia, or gastroparesis-like symptoms during therapy. Within the currently limited follow-up, clinical studies have not established a consistent overall increase in oesophageal cancer incidence, although subtype-specific findings are heterogeneous [8,9,10].

In practice, these findings should be interpreted as reassuring but not practice-changing. They do not justify changing Barrett’s oesophagus surveillance intervals, altering standard reflux management, or using incretin therapy as oesophageal chemoprevention [33,34,35,36,37]. Moreover, they do not remove the need to evaluate and treat new or worsening reflux, regurgitation, dysphagia, or gastroparesis-like symptoms during treatment with GLP-1R or dual GIP/GLP-1R agonists.

The primary clinical hypothesis generated by this study is that any long-term oesophageal benefit of incretin therapy, if present, is more likely to depend on the magnitude and durability of weight loss than on glucose lowering [36,37]. Future pharmacoepidemiologic studies should incorporate baseline reflux and Barrett status, endoscopic surveillance intensity, drug class, dose escalation, treatment duration, treatment-emergent upper gastrointestinal symptoms, and achieved, time-updated weight loss. They should also analyse reflux disease, Barrett’s oesophagus, and oesophageal adenocarcinoma separately.

To our knowledge, this is the first drug-target Mendelian randomisation study to evaluate GLP-1R and *GIPR* cis-regulated expression in relation to gastro-oesophageal reflux and the Barrett’s oesophagus/oesophageal adenocarcinoma axis jointly. Several features strengthen the clinical interpretation of these findings. First, it addresses a timely safety question for a drug class now used at population scale: whether genetic perturbation of GLP-1R and GIPR targets is genetically associated with reflux and the Barrett’s oesophagus/oesophageal adenocarcinoma pathway. This question is difficult to answer in trials, which often have a limited duration for cancer outcomes, and in observational studies, which remain vulnerable to confounding by indication, surveillance patterns, and weight-loss-related differences between treated and untreated patients. By using germline, cis-regulated receptor expression as a drug-target proxy, this study provides complementary evidence that is less susceptible to reverse causation and conventional confounding.

Second, we deliberately kept the analysis conservative. A prespecified, publicly registered, gated workflow required positive-control validation and colocalisation before interpreting any receptor–outcome association as target specific. This framework helped prevent overinterpretation of the extreme OAC-only estimates and strengthened the credibility of the central null finding, which was further supported by an independently derived, functionally defined instrument (*GLP1R* rs10305492) that does not depend on expression data (Table S16). Another strength was separating two clinically distinct mechanisms that are often conflated in practice: a potential long-term benefit through durable weight loss and a potential short-term harm through delayed gastric emptying and reflux symptoms. The latter mechanism is not modelled by genetic proxies. Finally, although the triangulation was illustrative rather than systematic, we interpreted the genetic findings alongside evidence from trials, cohort studies, pharmacovigilance, and bariatric-surgery literature. This triangulation yielded a clinically testable hypothesis: any oesophageal benefit of incretin therapy, if present, would be expected to track the magnitude and durability of weight loss rather than glycaemic control.

Some limitations frame interpretation. The study was adequately powered for reflux disease but underpowered for BO/OAC and OAC-only outcomes, so null cancer findings represent absence of evidence rather than evidence of absence; the combined BO/OAC endpoint is bounded by the few available adenocarcinoma cases, and an adequately powered evaluation will require larger, adenocarcinoma-specific, ancestry-diverse GWAS. As expected for drug-target cis-MR, receptor instruments comprised a single or a few cis-eQTLs, precluding many-variant pleiotropy-robust estimators; potential eQTLGen-outcome overlap could not be fully excluded, although strong instruments make substantial overlap bias unlikely, and the independent FinnGen replication (Table 3) further mitigates this concern. GIPR inference was constrained, as the GIPR-BMI and GIPR-type 2 diabetes positive-control estimates were unavailable and GIPR-reflux disease was not estimable, weakening evaluation of the GIP component of dual agonists such as tirzepatide; regional colocalisation nonetheless showed that the *GIPR* cis-eQTL shares a causal variant with type 2 diabetes (Table S18), which partly offsets this. Because the *GIPR* locus carries multiple independent metabolic association signals, and because coloc.abf assumes a single causal variant per locus, multi-signal colocalisation would be required to characterise it fully. The direction of GIPR perturbation is also not straightforward, since both higher expression and reduced-function coding variation align with a metabolically favourable profile. Finally, germline proxies model lifelong receptor expression and do not capture dose, duration, receptor occupancy, delayed gastric emptying, or treatment-emergent reflux; blood eQTLs may not fully represent oesophageal biology; and because multivariable MR is not identifiable with single-instrument receptors, the BMI-linked indirect estimate is only directional and hypothesis generating (Table S15).

## 5. Conclusions

In this genetic assessment of target-mediated oesophageal safety, genetically proxied GLP-1R perturbation was not associated with reflux disease (the GIPR-reflux disease estimate was not estimable), and no target-specific association with Barrett’s oesophagus/adenocarcinoma was demonstrated for either receptor. Colocalisation, however, was uninformative for these endpoints because the outcome loci carried no detectable association signal, so it neither supports nor excludes a shared causal variant. The null reflux association was reasonably precise and argues against a substantial target-mediated reflux liability, whereas limited precision, together with uninformative colocalisation, precluded exclusion of moderate effects for Barrett’s oesophagus and oesophageal adenocarcinoma. In pathway analyses, adiposity showed the most consistent metabolic association, whereas glycaemic traits did not, a contrast that persisted when HbA1c was instrumented from a substantially better-powered population-based dataset. These findings offer cautious genetic reassurance but do not establish long-term clinical safety, capture treatment-emergent reflux or dose effects, or justify changes to reflux management or Barrett’s surveillance. More definitive evaluation will require larger Barrett’s oesophagus/adenocarcinoma genome-wide association studies and pharmacoepidemiological or clinical cohort studies of incretin-based therapies.

## Figures and Tables

**Table 1:** Genetic data sources.

Trait (Role)	Dataset/Accession	Ancestry	Cases/Controls (or N)	Genome Build
GLP-1R, GIPR expression (exposure)	eQTLGen blood cis-eQTL	European	*N* = 31,684	GRCh37
Reflux disease (primary)	Ong et al. 2022 MTAG (GCST90000514) [14]	European	129,080/473,524 *	GRCh37
BO/OAC combined (primary)	Schroder et al. 2023; GCST90473003 [15]	European	16,790/32,476	GRCh37
OAC only (sensitivity)	Schroder et al. 2023; GCST90473005 [15]	European	240/12,534	GRCh37
ESCC (descriptive comparator)	GCST90271956 (trans-ancestry meta) [16]	East Asian and South African	2013/2701 (EAS)	GRCh37
BMI, HbA1c, type 2 diabetes (metabolic pathway traits)	ieu-b-40/ieu-b-4842/ebi-a-GCST006867	European	Large European GWAS datasets [17,18,19]	GRCh37

* Reflux disease case/control counts are the nominal counts from the source multitrait (MTAG) analysis (Ong et al. 2022 [14]); a priori power (Table S9) was computed from these counts. OAC-only (GCST90473005) included 240 adenocarcinoma cases and 12,534 controls (European; Schroder et al. 2023 [15]), contributing to limited precision in the sensitivity analyses. ESCC is trans-ancestry (East Asian and South African); the case/control counts (2013/2701) are from the East Asian (EAS) subset, and this dataset was used as a descriptive comparator given ancestry mismatch and limited power. Abbreviations: BMI, body mass index; BO, Barrett’s oesophagus; cis-eQTL, cis-expression quantitative trait locus; EAS, East Asian; ESCC, oesophageal squamous cell carcinoma; GIPR, glucose-dependent insulinotropic polypeptide receptor; GLP-1R, glucagon-like peptide-1 receptor; GWAS, genome-wide association study; HbA1c, glycated haemoglobin; OAC, oesophageal adenocarcinoma.

**Table 2:** Drug-target MR estimates, colocalisation, and clinical interpretation.

Target	Outcome	OR (95% CI)	*p* Value	PP.H4	Clinical Interpretation
GLP-1R	Reflux disease	0.97 (0.85–1.11)	0.70	0.004	No evidence of substantial target-mediated reflux liability
GIPR	Reflux disease	Not estimable	-	0.002 *	Regional PP.H4 only; no harmonised MR estimate
GLP-1R	BO/OAC	0.96 (0.47–1.96)	0.91	0.017	No target-specific signal; colocalisation uninformative
GLP-1R	BO/OAC (correlated instrument)	1.04 (0.56–1.94)	0.91	0.017	Sensitivity analysis; no target-specific signal; colocalisation uninformative
GIPR	BO/OAC	1.00 (0.42–2.37)	0.99	0.015	No target-specific signal; colocalisation uninformative
GLP-1R	OAC only	0.02 (0.001–0.41)	0.01	0.307	Extreme underpowered estimate; not interpreted as protective
GIPR	OAC only	0.02 (0.001–0.35)	0.008	0.430	Extreme underpowered estimate; not interpreted as protective
GIPR	ESCC	1.35 (0.79–2.31)	0.27	0.017	Descriptive comparator

Estimates are scaled to genetically proxied higher cis-regulated receptor expression as defined by the eQTL instrument and should not be interpreted as the effect of a specific drug dose or duration. PP.H4 is the posterior probability of a shared causal variant (colocalisation); the prespecified default-prior threshold for a target-specific interpretation was PP.H4 ≥ 0.70, which no pair met; for every pair except GIPR-reflux disease, the outcome locus carried no detectable association signal, so non-colocalisation is uninformative rather than evidence against a shared causal variant; at the *GIPR* locus, where the outcome signal is detectable, colocalisation indicates distinct causal variants (Table S17). * For GIPR-reflux disease, PP.H4 is a descriptive regional value; no harmonised MR estimate was derived because the cis-eQTL instrument was unavailable in the reflux disease GWAS. OAC-only rows are underpowered single-variant sensitivity analyses and were not interpreted as target specific. Full colocalisation results are in Table S11. Abbreviations: BO/OAC, Barrett’s oesophagus/oesophageal adenocarcinoma; CI, confidence interval; ESCC, oesophageal squamous cell carcinoma; GIPR, glucose-dependent insulinotropic polypeptide receptor; GLP-1R, glucagon-like peptide-1 receptor; MR, Mendelian randomisation; OAC, oesophageal adenocarcinoma; OR, odds ratio.

**Table 3:** Independent-population replication in FinnGen R12.

Target	Instrument Set	Outcome	OR (95% CI)	p Value	PP.H4
GLP-1R	Primary	Reflux disease	0.89 (0.72–1.09)	0.25	0.010
GLP-1R	Correlated	Reflux disease	0.94 (0.83–1.07)	0.33	0.010
GIPR	Primary	Reflux disease	0.99 (0.78–1.27)	0.96	0.005
GLP-1R	Primary	Barrett’s oesophagus	2.11 (0.86–5.20)	0.11	0.074
GLP-1R	Correlated	Barrett’s oesophagus	1.66 (0.95–2.93)	0.08	0.074
GIPR	Primary	Barrett’s oesophagus	0.49 (0.16–1.46)	0.20	0.039
GLP-1R	Primary	Oesophageal cancer	1.04 (0.36–2.94)	0.95	0.024
GLP-1R	Correlated	Oesophageal cancer	0.67 (0.34–1.29)	0.23	0.024
GIPR	Primary	Oesophageal cancer	3.71 (1.05–13.11)	0.04	0.127

Independent replication using FinnGen R12 (Finnish; 500,348 individuals), a population independent of the UK Biobank embedded in the reflux disease GWAS, addressing potential sample overlap. Odds ratios are per SD higher genetically proxied receptor expression from the same cis-expression instruments, harmonised to FinnGen by rs identifier; the primary instrument used the Wald ratio and the correlated set used correlation-aware inverse-variance weighting. PP.H4 is the colocalisation posterior probability of a shared causal variant for each receptor locus and outcome. FinnGen endpoints were K11_REFLUX (reflux disease), K11_BARRET (Barrett’s oesophagus) and C3_OESOPHAGUS_EXALLC (all oesophageal cancer, not adenocarcinoma-specific; 1277 cases). Estimates were consistent with the null for reflux disease with no colocalisation, reproducing the primary analysis; the nominal GIPR-oesophageal cancer estimate rested on a single variant with a very wide confidence interval and did not colocalise. CI, confidence interval; OR, odds ratio; PP.H4, posterior probability of a shared causal variant; SD, standard deviation. GIPR, glucose-dependent insulinotropic polypeptide receptor; GLP-1R, glucagon-like peptide-1 receptor.

## Data Availability

The data presented in this study are openly available in public repositories. Summary-level genome-wide association statistics were obtained from the GWAS Catalog (https://www.ebi.ac.uk/gwas/, accessed on 26 June 2026; accessions GCST90000514, GCST90473003, GCST90473005, GCST90271956) and IEU OpenGWAS (https://gwas.mrcieu.ac.uk/, accessed on 26 June 2026; ieu-b-40, ieu-b-4842, ebi-a-GCST006867, ebi-a-GCST90014006). Cis-expression quantitative trait loci were obtained from the eQTLGen Consortium (https://www.eqtlgen.org/, accessed on 26 June 2026) and GTEx v8 through the eQTL Catalogue (https://gtexportal.org/, accessed on 26 June 2026). Replication analyses used FinnGen release R12 (https://www.finngen.fi/en/access_results, accessed on 13 July 2026). The pre-registration, including the analysis plan, the positive-control direction gate and the colocalisation criterion, together with its dated updates, is publicly available at https://doi.org/10.17605/OSF.IO/FRVY8 (accessed on 18 August 2026). Full dataset details, accessions, and sample sizes are provided in Table 1 and Supplementary Material S1. Receptor expression levels quoted in the Methods were obtained from GTEx v10 (https://gtexportal.org/, accessed on 18 August 2026). No individual-level data were used. Analysis code is available from the corresponding author on reasonable request.

## Supplementary Materials

The following supporting information can be downloaded at https://www.mdpi.com/article/10.3390/biomedicines14091887/s1: Supplementary Material S1: Supplementary Methods; Figure S1: Analytic pipeline and prespecified decision gates; Figure S2: Assumed causal structure and identification assumptions (directed acyclic graph); Figure S3: Instrument strength (mean F); Figure S4: Positive-control direction gate; Figure S5: A priori power by outcome; Figure S6: Summary of drug-target MR estimates by receptor–outcome pair. Odds ratios (95% CI) with *p* values are shown; no receptor–outcome pair reached the colocalisation threshold (all PP.H4 <0.70), but colocalisation was uninformative for these endpoints because the outcome loci carried no detectable association signal (Table S17); Figure S7: Exploratory GLP-1R-BMI two-step pathway estimate; Figure S8: Metabolic trait-to-outcome MR estimates (path b); Figure S9: Descriptive comparison of the adenocarcinoma-axis outcome (BO/OAC) and the squamous cell comparator (ESCC); Figure S10: Tissue-specific GLP-1R cis-eQTL sensitivity analysis; Figure S11: Distinct time scales for reflux-related symptoms and weight-mediated oesophageal risk modification; Table S1: STROBE-MR reporting checklist (condensed); Table S2: Registered plan versus reported analyses, with rationale; Table S3: Genetic instruments for GLP-1R and *GIPR*; Table S4: SNP-level cis-eQTL instruments (eQTLGen whole blood, GRCh37); Table S5: Instrument availability by outcome after harmonisation; Table S6: Pairwise LD (r) among the correlated GLP-1R instruments (1000 Genomes EUR); Table S7: eQTLGen detection and testing metrics for *GLP1R* and GIPR; Table S8: Tissue-specific cis-eQTL availability (GTEx via the eQTL Catalogue); Table S9: A priori power (odds ratio 1.2 per SD); Table S10: Positive-control direction gate; Table S11: Colocalisation posterior probabilities and threshold sensitivity; Table S12: Colocalisation PP.H4 sensitivity to the shared-variant prior (p12); Table S13: Two-step pathway estimates via metabolic traits (exploratory); Table S14: Metabolic trait → outcome effects (path b, inverse-variance weighted); Table S15: Pleiotropy-robust sensitivity analyses for BMI → outcome MR (path b); Table S16: Functional coding-variant instrument set; Table S17: Full colocalisation posterior decomposition and regional outcome-signal summary; Table S18: Colocalisation of each receptor cis-locus with metabolic traits; Table S19: Mediator-to-outcome and two-step estimates using a population-based HbA1c GWAS, and indirect and implied direct effects by path-b estimator; Table S20: Pre-registered secondary outcomes analysed post hoc in FinnGen R12; Table S21: Illustrative clinical and epidemiological evidence used for triangulation.
