# Supplementary material for: Genetic Assessment of Oesophageal Safety of GLP-1 and GIP Receptor Perturbation: A Drug-Target Mendelian Randomisation Study
Source: Biomedicines. 2026 Aug 24;14(9):1887. doi: 10.3390/biomedicines14091887 (PMC13604261; doi:10.3390/biomedicines14091887)
Supplement: Supplementary file 1 [file biomedicines-14-01887-s001.zip › biomedicines-4490212-supplementary.pdf]

Supplementary Materials

Genetic assessment of oesophageal safety of GLP-1 and GIP receptor perturbation: a drug-target Mendelian randomisation study

Contents

Supplementary Material S1. Supplementary Methods
Supplementary Tables
Table S1. STROBE-MR reporting checklist (condensed)
Table S2. Registered plan versus reported analyses, with rationale
Table S3. Genetic instruments for GLP-1R and GIPR
Table S4. SNP-level cis-eQTL instruments (eQTLGen whole blood, GRCh37)
Table S5. Instrument availability by outcome after harmonisation
Table S6. Pairwise LD (r) among the correlated GLP-1R instruments (1000 Genomes EUR)
Table S7. eQTLGen detection and testing metrics for *GLP1R* and GIPR
Table S8. Tissue-specific cis-eQTL availability (GTEx via the eQTL Catalogue)
Table S9. A priori power (odds ratio 1.2 per SD)
Table S10. Positive-control direction gate
Table S11. Colocalisation posterior probabilities and threshold sensitivity
Table S12. Colocalisation PP.H4 sensitivity to the shared-variant prior (p12)
Table S13. Two-step pathway estimates via metabolic traits (exploratory)
Table S14. Metabolic trait → outcome effects (path b, inverse-variance weighted)
Table S15. Pleiotropy-robust sensitivity analyses for BMI → outcome MR (path b)
Table S16. Functional coding-variant instrument set
Table S17. Full colocalisation posterior decomposition and regional outcome-signal summary
Table S18. Colocalisation of each receptor cis-locus with metabolic traits
Table S19. Mediator-to-outcome and two-step estimates using a population-based HbA1c GWAS, and indirect and implied direct effects by path-b estimator
Table S20. Pre-registered secondary outcomes analysed post hoc in FinnGen R12
Table S21. Illustrative clinical and epidemiological evidence used for triangulation
Supplementary Figures
Figure S1. Analytic pipeline and prespecified decision gates
Figure S2. Assumed causal structure and identification assumptions (directed acyclic graph)
Figure S3. Instrument strength (mean F)
Figure S4. Positive-control direction gate
Figure S5. A priori power by outcome
Figure S6. Summary of drug-target MR estimates by receptor-outcome pair. Odds ratios (95% CI) with P values are shown; no receptor-outcome pair reached the colocalisation threshold (all PP.H4 <0.70), but colocalisation was uninformative for these endpoints because the outcome loci carried no detectable association signal (Table S17)
Figure S7. Exploratory GLP-1R-BMI two-step pathway estimate
Figure S8. Metabolic trait-to-outcome MR estimates (path b)
Figure S9. Descriptive comparison of the adenocarcinoma-axis outcome (BO/OAC) and the squamous-cell comparator (ESCC)
Figure S10. Tissue-specific GLP-1R cis-eQTL sensitivity analysis
Figure S11. Distinct time scales for reflux-related symptoms and weight-mediated oesophageal risk modification

Supplementary Material S1. Supplementary Methods

Study design and reporting

We performed a 2-sample, summary-level drug-target Mendelian randomisation analysis following a prespecified gated analysis plan and reported in accordance with the STROBE-MR guideline. All analyses used publicly available summary-level genome-wide association study (GWAS) data; oesophageal outcome data were examined only after the positive-control direction gate passed, and key analytic decisions are documented in Table S2. The estimand was the association of genetically proxied cis-regulated receptor expression with oesophageal outcomes, not the effect of initiating, continuing, or prescribing any specific GLP-1R agonist or dual GIP/GLP-1R agonist. Outcome definitions, case ascertainment, and phenotype harmonisation followed the original GWAS publications. Instrument relevance was assessed through cis-eQTL strength and positive-control associations, and potential confounding by variants in linkage disequilibrium was addressed through colocalisation; because the instruments comprised single or few cis-variants, conventional many-instrument pleiotropy-robust estimators were not applicable. Potential sample overlap between eQTLGen and the outcome GWAS could not be fully excluded from summary-level data; because the cis-eQTL instruments were strong, substantial bias from overlap was considered unlikely, but this was retained as a consideration in interpretation.

Genetic instruments

Instruments were cis-expression quantitative trait loci (cis-eQTLs) for *GLP1R* and *GIPR* messenger RNA expression from eQTLGen whole blood (n = 31,684), chosen as the primary source because it is the largest available cis-eQTL resource. Z scores were converted to beta coefficients and standard errors using the assessed-allele frequency and per-SNP sample size (Zhu approximation). We defined a 100-kb cis-window (genome build GRCh37) and retained genome-wide-significant variants (P < 5×10^-8). A primary instrument set used independent variants (clumping at $r^2 < 0.001$); a sensitivity set used correlated variants ($r^2 < 0.1$) with a signed linkage-disequilibrium matrix for correlation-aware estimation, using a 1000 Genomes European reference panel. Instruments were strong (mean F, 77.2 and 56.8) but comprised a single variant (primary) or a small correlated set, which is intrinsic to drug-target cis-MR of a single gene and precludes pleiotropy-robust estimators (MR-Egger, weighted median, MR-PRESSO) that require many independent variants. Effect alleles were oriented to higher receptor expression; throughout, the agonism direction denotes alignment with the metabolic effects expected from agonist therapy in the positive-control analyses and does not imply pharmacologic equivalence. Because the targets are membrane receptors, plasma cis-protein quantitative trait loci were not used.

Positive-control direction gate and power

Before any oesophageal outcome was examined, each instrument was tested against metabolic positive controls (type 2 diabetes, BMI, HbA1c). The direction gate required concordance with the expected agonist direction in at least 1 established positive control, with no control showing a directionally inconsistent estimate; statistical significance was not required for every control because cis-eQTL instruments for a single receptor are few. A priori power was computed per outcome using the Burgess noncentrality approach.

Colocalisation

To distinguish a target-specific effect from confounding by variants in linkage disequilibrium, we performed Bayesian colocalisation (coloc.abf) across each cis-window using default priors (p1 = p2 = 1×10^-4; p12 = 1×10^-5), requiring a posterior probability of a shared causal variant (PP.H4) of at least 0.70 as a prespecified, pragmatic robustness criterion. We verified that no conclusion changed at stricter thresholds (0.80 and 0.90) or when the shared-variant prior p12 was varied from 1×10^-6 to 1×10^-4 (Tables S11 and 12). Colocalisation assumes a single causal variant per locus, and its power is constrained by outcome-locus power.

Two-step pathway analysis

We performed a prespecified 2-step (product-of-coefficients) pathway analysis, in which the receptor-to-mediator effect (from the direction gate) is multiplied by the mediator-to-outcome effect (independent inverse-variance weighted MR) to give the indirect effect, with a delta-method standard error. Because a multivariable MR is not identifiable with single-instrument receptors, this approach was used to identify which metabolic pathway is most consistently linked to the outcomes rather than to establish an overall receptor effect; indirect estimates are reported as nominal and hypothesis-generating. Pleiotropy-robust sensitivity analyses for the mediator-to-outcome estimates are provided in Table S15.

Tissue-specific sensitivity and software

To address tissue specificity, the analysis was repeated using tissue cis-eQTLs for pancreas, small intestine, stomach, oesophageal mucosa, hypothalamus, and adipose tissue, obtained from GTEx through the eQTL Catalogue; because GTEx tissue sample sizes are small, suggestive lead cis-eQTLs (P < 1×10^-5) were used only for sensitivity analyses. Analyses used R version 4.5.3 with the TwoSampleMR (version 0.7.4), MendelianRandomisation (version 0.10.0), coloc (version 5.2.3), and ieugwasr (version 1.1.0) packages, which were the most recent releases at the time of analysis.

Independent-population replication (FinnGen R12)

To assess whether the primary null findings could be explained by sample overlap between eQTLGen and the UK Biobank component of the Ong et al. 2022 reflux disease MTAG, we repeated the drug-target

cis-MR and colocalisation using FinnGen R12 (Finnish; 500,348 individuals; genome build GRCh38) as the outcome source, a population independent of the UK Biobank. The same eQTLGen cis-eQTL instruments for *GLP1R* and *GIPR* were used, harmonised to FinnGen by rsID; outcomes were gastro-oesophageal reflux (K11_REFLUX), Barrett's oesophagus (K11_BARRET), and oesophageal cancer (C3_OESOPHAGUS_EXALLC; all oesophageal cancer, not adenocarcinoma-specific). MR used the Wald ratio (primary instrument) or correlation-aware inverse-variance weighting (correlated set), and colocalisation used coloc.abf with default priors within each cis-window (Table 3 in the main article).

Supplementary Tables

Table S1. STROBE-MR reporting checklist (condensed).

#	STROBE-MR item	Reported	Location
1	Title/abstract: MR design stated	Yes	Title; Abstract
2	Background and rationale	Yes	Introduction
3	Specific objectives and hypothesis	Yes	Introduction (final paragraph)
4	Study design and data sources	Yes	Methods; Table 1
5	Exposure, outcome, mediators defined	Yes	Methods; Table 1
6	Instrument selection and assumptions	Yes	Methods (instruments)
7	Assessment of instrument strength (F, R^2)	Yes	Methods; Table S3
8	Assessment of relevance / validity (positive controls)	Yes	Methods; Table S10
9	Statistical methods and MR estimator	Yes	Methods
10	Sensitivity analyses (correlated IVW, tissue, colocalisation)	Yes	Methods; Results; appendix
11	Assessment of linked-variant confounding / target specificity (colocalisation)	Yes	Methods; Results; Table 2
12	Power / precision	Yes	Methods; Table S9
13	Descriptive data / instruments	Yes	Tables S3–S6 and S8
14	Main results with CIs	Yes	Table 2; Figure 2
15	Sensitivity/pathway results	Yes	Figure 3; Tables S13–S15
16	Key results and interpretation	Yes	Discussion
17	Limitations (power, single IV, tissue, pleiotropy)	Yes	Discussion
18	Generalisability (ancestry)	Yes	Discussion
19	Data/code availability	Yes	Methods; Supplement
20	Funding	Yes	Article Information

Condensed mapping to the STROBE-MR checklist (Skrivankova et al. 2021). Complete the official checklist form at submission.

Table S2. Registered plan versus reported analyses, with rationale.

No.	Item	Initial plan	Final approach and rationale
1	reflux disease outcome dataset	Ong et al. 2019 (GCST008832; lead SNPs only)	Changed to Ong et al. 2022 MTAG (GCST90000514), the largest European reflux disease GWAS with full summary statistics, because the initially planned source lacked genome-wide statistics containing the cis-instrument variants. Same trait and ancestry; increases power.
2	BO/OAC statistical power	Expected adequate power	Realized power for BO/OAC was low (0.13-0.17) with the single-instrument primary analysis; retained as primary but reported as underpowered, with power recovered partly via the correlated-instrument analysis. No change to hypotheses or outcomes.
3	Pathway estimator	MVMR + product-of-coefficients	Product-of-coefficients (two-step) analysis was performed. Because MVMR was not identifiable with single-instrument receptor proxies, the analysis was limited to product-of-coefficients pathway consistency estimates, and direct effects were not interpreted. Same hypotheses, pathway traits, and outcomes.
4	Registered secondary outcomes (gastric cancer, peptic ulcer disease, chronic gastritis)	Evaluated as drug-safety outcomes	A priori power was below 0.17 for both receptors at the pre-analysis power assessment (26 June 2026, before any oesophageal outcome data were examined), so these outcomes were designated exploratory and were not analysed for the original report. They were completed during revision in FinnGen R12, ancestry-matched to the European instruments, and are reported irrespective of direction (Table S20).
5	Gastroparesis / upper-gastrointestinal dysmotility	Exploratory analysis if a suitable GWAS became available	Not analysable: no adequately powered genome-wide association study exists. Identified at the design stage.
6	Colocalisation estimator	coloc and SuSiE	coloc.abf only, with prior and threshold sensitivity analyses. Each receptor locus yielded a single cis-eQTL instrument or a small correlated set with no evidence of multiple independent cis-signals, so multi-signal colocalisation was not applied. The prespecified PP.H4 >= 0.70 criterion is unchanged. Full posterior decompositions are now reported (Table S17).
7	Replication population	FinnGen and BioBank Japan	FinnGen R12 only. This addresses the principal threat, sample overlap with the UK Biobank component of the reflux disease GWAS, within the same ancestry as the European instruments. BioBank Japan replication was not attempted because European-derived instruments are not ancestry-matched to East Asian outcome data. The East Asian ESCC dataset was retained as a descriptive comparator, as registered.
8	Attribution of the BO/OAC dataset	Recorded at registration as Bonfiglio et al. 2022 (16,790 cases / 32,476 controls)	Author-attribution error at registration. The dataset used, as registered by sample size and design, is Schroder et al. (Gut 2023; GWAS Catalog GCST90473003), and it is cited as such

No.	Item	Initial plan	Final approach and rationale
			throughout. The dataset itself is unchanged.
9	Analyses added in response to peer review	Not applicable	Four analyses were added during revision and pre-committed in a dated registration update before they were run: a functional coding-variant instrument set (Table S16); colocalisation of each receptor cis-locus with BMI, HbA1c and type 2 diabetes (Table S18); repetition of the mediator-to-outcome and two-step analyses using a population-based HbA1c GWAS (Table S19); and eQTLGen detection and testing metrics (Table S7).

The study was prospectively registered (https://doi.org/10.17605/OSF.IO/FRVY8; registered 24 June 2026). Items 1 to 3 were decided during the study, before any oesophageal outcome estimate was examined, and were posted as a dated registration update on 26 June 2026. Items 4 to 9 complete the comparison between the registered plan and the analyses reported here, and were posted as a second dated registration update on 18 August 2026, before the additional analyses were run. Together these account for all differences between the registered plan and this report.

Table S3. Genetic instruments for GLP-1R and GIPR.

Receptor	Instrument set	No. of variants	Mean F statistic	Total R^2
GLP-1R	primary (r^2<0.001)	1	77.2	0.0026
GLP-1R	correlated (r^2<0.1 + LD)	3	52.7	0.0163
GIPR	primary (r^2<0.001)	1	56.8	0.0018
GIPR	correlated (r^2<0.1 + LD)	1	56.8	0.0018

cis-eQTL instruments (eQTLGen blood). F statistics well above the conventional weak-instrument threshold of 10 are consistent with a low risk of weak-instrument bias, which in a two-sample design acts towards the null. F indexes the precision of the SNP-exposure association and does not indicate that a variant is biologically influential; the variance in receptor expression explained is small (0.26% for GLP1R and 0.18% for GIPR). The correlated set adds independent signal for GLP-1R only. Single/few instruments preclude MR-Egger, weighted median and MR-PRESSO. The correlated GLP-1R set contained three cis-eQTLs before outcome harmonisation; the number contributing to each MR estimate (e.g. two for BO/OAC in Table 2) varied with availability and harmonisation in the corresponding outcome GWAS.

Table S4. SNP-level cis-eQTL instruments (eQTLGen whole blood, GRCh37).

Receptor	Instrument set	SNP	Chromosome:position	Effect/other allele	Effect-allele frequency	β (SE)	P Value	F statistic
GLP-1R	primary	rs9283907	6:39026703	A/G	0.151	0.101 (0.012)	1.5×10^-18	77.2
GIPR	primary	rs12972094	19:46165604	A/C	0.239	-0.071 (0.009)	4.8×10^-14	56.8
GLP-1R	correlated	rs114977861	6:38971093	C/T	0.013	0.694 (0.109)	2.1×10^-10	40.4
GLP-1R	correlated	rs10305420	6:39016636	T/C	0.364	0.055 (0.009)	1.9×10^-10	40.5
GLP-1R	correlated	rs9283907	6:39026703	A/G	0.151	0.101 (0.012)	1.5×10^-18	77.2
GIPR	correlated	rs12972094	19:46165604	A/C	0.239	-0.071 (0.009)	4.8×10^-14	56.8

EA/OA, effect/other allele; EAF, effect-allele frequency. Betas/SEs are shown as reported by eQTLGen on the expression scale; the MR analyses re-oriented alleles so that a positive exposure effect corresponded to higher receptor expression. The GIPR correlated set is identical to its single primary instrument (all candidate variants were in LD $r^2>0.1$ with the lead).

Table S5. Instrument availability by outcome after harmonisation.

Outcome	GLP-1R primary	GLP-1R correlated (retained)	GIPR primary
reflux disease	available	1 of 3	not available
BO/OAC	available	2 of 3	available
OAC only	available	2 of 3	available
ESCC	not available (ancestry)	not applicable	available

Availability reflects presence of the cis-eQTL SNP in each outcome GWAS after harmonisation; no LD-proxy substitution was used for MR estimation, and proxy searches (r^2>0.8) served only to confirm absence or regional context. GIPR was absent from the reflux disease GWAS (MTAG SNP set), so GIPR→reflux disease was not estimable; GLP-1R was absent from the East-Asian/South-African ESCC meta-analysis (ancestry mismatch). We confirmed in the full Ong et al. 2022 reflux disease summary statistics that neither rs12972094 nor its clumped correlated variants were present in the reflux disease SNP set, so no GIPR-reflux disease estimate was possible in this dataset. No proxy substitution was performed, to avoid introducing proxy-related ambiguity in a single-instrument cis-MR setting. For GLP-1R and ESCC, no harmonised Wald MR estimate was possible (ancestry mismatch), so ESCC does not appear in the GLP-1R MR forest; the GLP-1R→ESCC entries in Tables S11 and 12 are regional colocalisation results at the cis-locus, which remain computable from the overlapping variants and are reported only as descriptive comparators.

Table S6. Pairwise LD (r) among the correlated GLP-1R instruments (1000 Genomes EUR).

SNP	rs114977861	rs10305420	rs9283907
rs114977861	1.00	0.14	0.26
rs10305420	0.14	1.00	0.21
rs9283907	0.26	0.21	1.00

Signed LD correlation matrix used for correlation-aware inverse-variance weighted estimation.

Table S7. eQTLGen detection and testing metrics for *GLP1R* and GIPR.

**Metric**	***GLP1R***	**GIPR**
Ensembl gene	ENSG00000112164	ENSG00000010310
cis-variants tested (whole file)	7692	7443
cis-variants tested in the 100-kb window	1025	666
variants with P < 5x10-8 in the window	28	77
variants with FDR < 0.05 in the window	65	103
median NrSamples	29,494	27,096
maximum NrSamples	29,623	31,470
eQTLGen total sample size	31,684	31,684
detection fraction, maximum NrSamples / total	0.935	0.993
detection fraction, median NrSamples / total	0.931	0.855
lead cis-eQTL	rs9283907	rs12972094
lead cis-eQTL P value	1.5x10-18	4.8x10-14
lead cis-eQTL F statistic	77.2	56.8
GTEx v10 whole-blood median TPM	0.031	1.012
GTEx v10 tissue with highest expression (median TPM)	Pancreas (about 6)	Adipose and lung (over 10)

eQTLGen reports association statistics rather than expression levels, so detection is indexed by NrSamples, the number of samples in which the variant was genotyped and the transcript measured; this reflects transcript detection together with cohort inclusion. Both the maximum-based and the median-based fraction are given, because NrSamples varies across variants within a locus. Whole-blood expression is low for *GLP1R* in particular, but both transcripts were quantified in most of the eQTLGen sample and yielded multiple genome-wide-significant cis-eQTLs. TPM, transcripts per million.

Table S8. Tissue-specific cis-eQTL availability (GTEx via the eQTL Catalogue).

Tissue	GLP-1R cis-eQTL	*GIPR* cis-eQTL	Note
Pancreas	Yes (rs34242937, F statistic 20.4)	No	Used for sensitivity; BO/OAC OR 1.02 (0.86-1.21), concordant with blood
Oesophageal mucosa	No	No	No suggestive lead cis-eQTL (P < 1×10^-5)
Stomach	No	No	No suggestive lead cis-eQTL (P < 1×10^-5)
Small intestine	No	No	No suggestive lead cis-eQTL (P < 1×10^-5)
Adipose (subcutaneous)	No	No	Low expression
Hypothalamus	No	No	Limited sample size

GTEx tissues queried via the eQTL Catalogue (lead-variant P < 1×10^-5). Only pancreatic GLP-1R yielded a usable cis-instrument; no usable cis-eQTL was detected for GIPR in any queried tissue, consistent with low expression and limited tissue eQTL power; tissue-level corroboration of the blood-based instrument is therefore available for GLP-1R only. This p<1×10^-5 threshold is less stringent than the primary eQTLGen threshold and was used only for tissue sensitivity. GTEx tissue sample sizes are smaller than eQTLGen, so absence of a cis-eQTL should be interpreted cautiously rather than as evidence of no regulation.

Table S9. A priori power (odds ratio 1.2 per SD).

Outcome	Role	Power GLP-1R	Power GIPR	Minimum detectable OR, GLP-1R/GIPR (80% power)
reflux disease	primary (single instrument)	0.85	not estimable†	1.19 / NE†
BO/OAC	primary (single instrument)	0.17	0.13	1.68 / 1.87
BO/OAC	correlated GLP-1R set	0.20	not applicable	1.58 / -
ESCC	descriptive comparator	0.06	0.06	5.0 / NE

Burgess et al. (2014) non-centrality approach; power to detect OR 1.2 per SD of genetically proxied expression. Primary rows use the single cis-instrument (sum R^2: GLP-1R 0.0026, GIPR 0.0018). Of the three-variant GLP-1R correlated set, only two variants (rs9283907 and rs10305420) were available in the BO/OAC GWAS after harmonisation (the rare rs114977861 was absent); their linkage-disequilibrium-adjusted joint R^2 (about 0.0034) provided only limited BO/OAC power (about 0.20, minimum detectable OR about 1.58). GIPR is a single instrument for all outcomes. NE, not estimable. †GIPR-reflux disease was not estimable; theoretical values (power 0.70 and minimum detectable OR 1.23, conditional on instrument availability) would apply, but no harmonised GIPR-reflux disease MR estimate was derived because the cis-eQTL instrument was unavailable in the reflux disease GWAS. Adequately powered only for the estimable GLP-1R-reflux disease analysis; the BO/OAC endpoints remain underpowered (absence of evidence, not evidence of absence).

Table S10. Positive-control direction gate.

Receptor	Positive control	β (per higher expression)	P Value	Direction consistent
GLP-1R	T2D	-0.088	0.41	Yes
GLP-1R	BMI	-0.081	0.0016	Yes
GLP-1R	HbA1c	-0.022	0.80	Yes
GIPR	HbA1c	-0.403	0.00018	Yes
GIPR	BMI	Unavailable	-	Not applicable
GIPR	T2D	Unavailable	-	Not applicable

Negative effects indicate alignment with the expected agonism direction in the positive-control analyses. GIPR→T2D and GIPR→BMI were unavailable (proxy not found). Gate PASS for both receptors.

Table S11. Colocalisation posterior probabilities and threshold sensitivity.

Exposure	Outcome	PP.H4	Colocalizes (≥0.70)	Colocalizes (≥0.80)	Colocalizes (≥0.90)
GLP-1R	reflux disease	0.004	No	No	No
GIPR	reflux disease	0.002	No	No	No
GLP-1R	BO/OAC	0.017	No	No	No
GIPR	BO/OAC	0.015	No	No	No
GLP-1R	OAC only	0.307	No	No	No
GIPR	OAC only	0.430	No	No	No
GLP-1R	ESCC	0.035	No	No	No
GIPR	ESCC	0.017	No	No	No

coloc.abf with default priors (p1=p2=1×10^-4, p12=1×10^-5). Under the default-prior analysis, no receptor-outcome pair reached the shared-variant posterior threshold at 0.70, 0.80 or 0.90, so the conclusion is unchanged across thresholds. Where the outcome locus carried no independent signal, posterior mass concentrated on PP.H1 (association with the eQTL only): for the ESCC comparators the full decomposition was GLP-1R H0<0.001, H1=0.953, H2<0.001, H3=0.011, H4=0.035 and GIPR H1=0.971, H3=0.011, H4=0.017; for GIPR→reflux disease posterior mass fell on PP.H3 (distinct causal variants; H3=0.79, H4=0.002). Because the outcome loci carry no detectable association signal, colocalisation is uninformative for these pairs rather than negative: non-colocalisation neither supports nor excludes a shared causal variant. Full posterior decompositions and regional signal summaries are given in Table S17. coloc.abf assumes a single causal variant per locus; multi-signal or fine-mapping-based colocalisation was not performed given the single or few cis-instruments available, so results are interpreted as robustness evidence rather than definitive proof.

Table S12. Colocalisation PP.H4 sensitivity to the shared-variant prior (p12).

Exposure	Outcome	p12=1×10^-6	p12=1×10^-5 (default)	p12=1×10^-4	Colocalizes at any prior
GLP-1R	reflux disease	<0.001	0.004	0.039	No
GIPR	reflux disease	<0.001	0.002	0.020	No
GLP-1R	BO/OAC	0.002	0.017	0.147	No
GIPR	BO/OAC	0.002	0.015	0.132	No
GLP-1R	OAC only	0.042	0.307	0.816	Permissive prior only; not interpreted
GIPR	OAC only	0.070	0.430	0.883	Permissive prior only; not interpreted
GLP-1R	ESCC	0.004	0.035	0.269	No
GIPR	ESCC	0.002	0.017	0.151	No

PP.H4 recomputed while varying the shared-variant prior p12 and holding p1=p2=1×10^-4. Because only the H4 prior depends on p12, PP.H4(p12') = k × PP.H4 / ((1 - PP.H4) + k × PP.H4) with k = p12'/p12; this is exact (validated against the full ESCC decomposition). Excluding the underpowered OAC-only sensitivity rows, no primary, co-primary, or descriptive-comparator pair reached the threshold at any prior, including the permissive p12=1×10^-4 setting (all PP.H4 ≤0.27); as elsewhere, this is uninformative rather than negative where the outcome locus carries no signal (Table S17). The OAC-only sensitivity estimates exceeded 0.70 only under the permissive prior (0.816 and 0.883) but were single-variant, underpowered estimates and were not interpreted as target-specific.

Table S13. Two-step pathway estimates via metabolic traits (exploratory).

Receptor → pathway trait → outcome	a	b	Indirect a×b (95% CI)	P Value
GLP-1R → BMI → BO/OAC	-0.081	0.263	-0.021 (-0.040, -0.002)	0.029
GLP-1R → HbA1c → BO/OAC	-0.022	0.060	-0.001 (-0.012, 0.009)	0.81
GLP-1R → T2D → BO/OAC	-0.088	0.032	-0.003 (-0.013, 0.007)	0.58
GLP-1R → BMI → reflux disease	-0.081	0.758	-0.061 (-0.100, -0.023)	0.0017
GLP-1R → HbA1c → reflux disease	-0.022	0.052	-0.001 (-0.010, 0.008)	0.80
GLP-1R → T2D → reflux disease	-0.088	0.037	-0.003 (-0.011, 0.005)	0.43
GIPR → HbA1c → BO/OAC	-0.403	0.060	-0.024 (-0.084, 0.035)	0.42

Two-step product-of-coefficients estimates on the log-odds scale (negative = protective); 95% CI by the delta method. Here, a is the receptor-to-mediator effect, b is the mediator-to-outcome effect, and a×b is the indirect (pathway) effect. Only the BMI pathway excluded the null in nominal product-of-coefficients analyses; however, BMI→BO/OAC was less robust in pleiotropy-robust sensitivity analyses (Table S15). Proportion mediated and direct effects are not tabulated because they are unstable when the total effect is near zero; inference rests on the indirect estimate, which is hypothesis-generating given single-instrument receptors. P values are nominal and not corrected for the multiple pathway tests examined; because GIPR-BMI and GIPR-type 2 diabetes positive-control estimates were unavailable, BMI-mediated receptor-specific inference is primarily GLP-1R-based. The GLP-1R rows correspond to the estimates plotted in Figure 3B; GIPR-specific pathway analysis was limited to HbA1c because GIPR-BMI and GIPR-type 2 diabetes estimates were unavailable. See Table S15 for pleiotropy-robust sensitivity of the underlying path-b (pathway trait→outcome) estimates.

Table S14. Metabolic trait → outcome effects (path b, inverse-variance weighted).

Pathway trait	Outcome	No. of variants	β	P Value
BMI	reflux disease	481	0.758	3×10^-197
BMI	BO/OAC	485	0.263	0.0024
HbA1c	reflux disease	9	0.052	0.14
HbA1c	BO/OAC	29	0.060	0.41
T2D	reflux disease	102	0.037	0.006
T2D	BO/OAC	115	0.032	0.46

BMI showed the most consistent association with both reflux disease and BO/OAC; in pleiotropy-robust sensitivity analyses this was robust for reflux disease but IVW-only and less robust for BO/OAC (Table S15). HbA1c was not associated with either outcome, and type 2 diabetes was not associated with BO/OAC, although a small association with reflux disease was observed (p=0.006).

Table S15. Pleiotropy-robust sensitivity analyses for BMI → outcome MR (path b).

Statistic	BMI → reflux disease	BMI → BO/OAC
No. of variants	443	447
IVW β (P)	0.756 (<1×10^-100)	0.258 (0.004)
Weighted median β (P)	0.662 (<1×10^-100)	0.083 (0.59)
MR-Egger β (P)	0.494 (2×10^-13)	0.166 (0.47)
Egger intercept (P)	0.0047 (1.6×10-5)	0.0016 (0.66)
Cochran Q P value	<0.001	0.004
MR-PRESSO global test P value	0.001	0.003

Path-b sensitivity on the palindrome-free harmonised instrument set (~500 genome-wide BMI instruments, OpenGWAS ieu-b-40); IVW reproduces the primary path-b estimates (Table S14). For BMI → reflux disease the effect is directionally consistent and significant across IVW, weighted median and MR-Egger, with substantial heterogeneity (Cochran Q p<0.001; MR-PRESSO global p=0.001) and directional pleiotropy (MR-Egger intercept 0.0047, p=1.6×10^-5; the value of p=0.002 given in the original submission was a transcription error, corrected here from the same analysis output, with no change to the conclusion) as expected for a highly polygenic exposure; leave-one-out IVW estimates were stable (0.75-0.78). For BMI → BO/OAC the IVW association was not corroborated by weighted median (0.08, p=0.59) or MR-Egger (0.17, p=0.47), with no directional pleiotropy (intercept p=0.66), reinforcing the cautious, hypothesis-generating interpretation of the Barrett's/OAC pathway. Steiger directionality favoured exposure → outcome for BO/OAC; for the MTAG-based reflux disease outcome, per-variant r-squared is inflated, making the Steiger comparison uninformative.

Table S16. Functional coding-variant instrument set.

Receptor	Variant (protein change)	Aligned allele	Outcome	Beta (SE)	P value
GLP-1R	rs10305492 (p.Ala316Thr)	A (HbA1c-lowering)	Reflux disease	-0.0092 (0.0193)	0.63
GLP-1R	rs10305492 (p.Ala316Thr)	A (HbA1c-lowering)	BO/OAC	-0.0282 (0.102)	0.78
GLP-1R	rs10305492 (p.Ala316Thr)	A (HbA1c-lowering)	OAC only	-0.2425 (0.417)	0.56
GIPR	rs1800437 (p.Glu354Gln)	C (Gln354, reduced function)	Reflux disease	-0.0310 (0.0061)	3.6x10-7
GIPR	rs1800437 (p.Glu354Gln)	C (Gln354, reduced function)	BO/OAC	+0.0180 (0.0323)	0.58
GIPR	rs1800437 (p.Glu354Gln)	C (Gln354, reduced function)	OAC only	+0.0026 (0.117)	0.98
GIPR	rs1800437 (p.Glu354Gln)	C (Gln354, reduced function)	ESCC	-0.0015 (0.020)	0.94

Coding variants cannot be placed on the cis-regulated expression scale of the primary instrument and are therefore reported as a separate sensitivity set. The per-allele associations shown here are the result: they are aligned to the direction indicated, are unaffected by the choice of scaling trait, and require no anchor. Ratio (Wald) estimates scaled on a glycaemic anchor are deliberately not reported, because the available HbA1c anchor (ieu-b-4842, the dataset used for the direction gate) is weak for both variants: for rs10305492 the anchor is beta -0.0696 (SE 0.0260), P = 0.0074, F = 7.2, which is below the conventional threshold of 10, and for rs1800437 it is essentially null (P = 0.71, F < 1). A BMI-anchored ratio for rs1800437 is estimable (odds ratio 2.53, 95% CI 1.77-3.61 per SD higher BMI acting through the receptor) but recovers the BMI-to-reflux disease pathway rather than a receptor-specific effect, and is not interpreted as such. BO/OAC, Barrett's oesophagus/oesophageal adenocarcinoma; ESCC, oesophageal squamous cell carcinoma; OAC, oesophageal adenocarcinoma; SE, standard error.

Table S17. Full colocalisation posterior decomposition and regional outcome-signal summary.

Receptor	Outcome	No. of SNPs	PP.H0	PP.H1	PP.H2	PP.H3	PP.H4	PP.H4/(PP.H3+PP.H4)	Region minimum P	Region maximum -log10 P	Variants P<1x10-5
GLP-1R	Reflux disease	361	<0.001	0.994	<0.001	0.002	0.004	0.66	5.9x10-2	1.23	0
GLP-1R	BO/OAC	840	<0.001	0.950	<0.001	0.033	0.017	0.35	1.3x10-2	1.89	0
GLP-1R	OAC only	840	<0.001	0.655	<0.001	0.038	0.307	0.89	1.1x10-2	1.97	0
GLP-1R	ESCC	501	<0.001	0.953	<0.001	0.011	0.036	0.76	5.2x10-4	3.28	0
GIPR	Reflux disease	92	<0.001	0.206	<0.001	0.792	0.002	0.002	3.6x10-7	6.44	4
GIPR	BO/OAC	533	<0.001	0.961	<0.001	0.024	0.015	0.38	2.0x10-3	2.70	0
GIPR	OAC only	532	<0.001	0.531	<0.001	0.040	0.430	0.92	2.1x10-3	2.68	0
GIPR	ESCC	370	<0.001	0.971	<0.001	0.011	0.017	0.60	1.8x10-3	2.75	0

coloc.abf with default priors. For every pair except GIPR-reflux disease the outcome locus carries no detectable association (maximum -log10 P 1.2 to 3.3; no variant at P < 1x10-5) and posterior mass concentrates on PP.H1, association with the eQTL only; colocalisation is therefore uninformative rather than negative for these pairs. The conditional quantity PP.H4/(PP.H3+PP.H4) is shown as requested. It is unstable where PP.H3+PP.H4 is very small, which is the case for the co-primary and comparator pairs (0.006 to 0.050), and in those rows a high conditional value is computed from almost no posterior mass and should not be read as evidence of sharing. For the two underpowered OAC-only rows PP.H3+PP.H4 is larger (0.35 and 0.47), so the conditional value is better determined there, but those estimates rest on a single variant and are not interpreted as target-specific. GIPR-reflux disease is the one locus with a detectable outcome signal, and there colocalisation is informative and indicates distinct causal variants (PP.H3 = 0.79).

Table S18. Colocalisation of each receptor cis-locus with metabolic traits.

Receptor	Trait	No. of SNPs	PP.H1	PP.H3	PP.H4	Colocalises (PP.H4>=0.70)	Region maximum -log10 P	Variants P<5x10-8
GLP-1R	BMI	344	0.360	0.551	0.090	No	6.49	0
GLP-1R	HbA1c	773	0.931	0.063	0.006	No	3.77	0
GLP-1R	Type 2 diabetes	628	0.913	0.080	0.007	No	4.24	0
GIPR	BMI	85	<0.001	1.000	<0.001	No	59.77	13
GIPR	HbA1c	486	<0.001	0.978	0.022	No	9.54	42
GIPR	Type 2 diabetes	90	<0.001	0.010	0.990	Yes	15.51	26

The *GIPR* cis-eQTL shares a causal variant with the type 2 diabetes signal at this locus (PP.H4 = 0.99), indicating that the instrument tags receptor-relevant biology rather than a linked variant, and supplying the GIPR-type 2 diabetes validation that was not obtainable as an MR estimate because the instrument variant is absent from that dataset after harmonisation. This comparison rests on 90 overlapping variants, similar to BMI (85) but far fewer than HbA1c (486), and should be read with that in mind. The BMI signal at the same locus arises from a distinct causal variant (PP.H3 = 1.00), so BMI-related association in the *GIPR*-QPCTL region should not be attributed to *GIPR* expression. HbA1c also favours distinct variants, but the window contains 42 genome-wide-significant HbA1c variants, which violates the single-causal-variant assumption of coloc.abf and favours PP.H3; multi-signal colocalisation would be required to resolve this. At the *GLP1R* locus no metabolic trait reaches genome-wide significance, so colocalisation cannot adjudicate there.

Table S19. Panel A. Mediator-to-outcome and two-step estimates using a population-based HbA1c GWAS, and indirect and implied direct effects by path-b estimator.

Exposure	Outcome	No. of variants	IVW beta	IVW P	Weighted median P	MR-Egger P	Egger intercept P
HbA1c, population-based (UK Biobank)	Reflux disease	109	0.040	0.23	0.95	0.39	0.080
HbA1c, within-sibship (as published)	Reflux disease	9	0.052	0.14	0.048	0.16	0.31
BMI (as published)	Reflux disease	481	0.758	3x10-197	1x10-100	4x10-14	1.1x10-5
HbA1c, population-based (UK Biobank)	BO/OAC	333	0.055	0.42	0.90	0.93	0.63
HbA1c, within-sibship (as published)	BO/OAC	29	0.061	0.41	0.84	0.72	0.37
BMI (as published)	BO/OAC	485	0.263	0.0024	0.59	0.36	0.77

Panel A. With an order of magnitude more instruments from a population-based genome-wide association study, HbA1c remains unassociated with both co-primary outcomes, so the more consistent association of adiposity is not attributable to instrument number or to the within-sibship design. Two-step indirect estimates through population-based HbA1c were likewise null (GLP-1R: reflux disease -0.0030, P = 0.27; BO/OAC -0.0041, P = 0.44. GIPR: reflux disease -0.0047, P = 0.26; BO/OAC -0.0065, P = 0.43). The within-sibship dataset remains the source used for the pre-registered direction gate, which is unchanged. The BMI rows here were re-estimated alongside HbA1c so that all three exposures use the same pipeline, and therefore rest on 481 and 485 variants; Table S2 reports the same BMI analyses on the palindrome-free harmonised set (443 and 447 variants), which is why the pleiotropy statistics differ slightly between the two tables. IVW, inverse-variance weighted.

Table S19, Panel B. Indirect and implied direct effects for GLP-1R via BMI, by path-b estimator.

Outcome	Path-b estimator	b (BMI to outcome)	Indirect (95% CI)	Indirect P	Total	Implied direct (95% CI)	Implied direct P
Reflux disease	IVW	0.756	-0.061 (-0.099 to -0.023)	0.0017	-0.026	0.035 (-0.103 to 0.173)	0.62
Reflux disease	Weighted median	0.662	-0.054 (-0.087 to -0.020)	0.0018	-0.026	0.028 (-0.109 to 0.165)	0.69
Reflux disease	MR-Egger	0.494	-0.040 (-0.067 to -0.013)	0.0036	-0.026	0.014 (-0.122 to 0.149)	0.84
BO/OAC	IVW	0.258	-0.021 (-0.040 to -0.002)	0.034	-0.042	-0.021 (-0.734 to 0.692)	0.95
BO/OAC	Weighted median	0.083	-0.007 (-0.031 to 0.018)	0.59	-0.042	-0.035 (-0.748 to 0.678)	0.92
BO/OAC	MR-Egger	0.166	-0.013 (-0.051 to 0.024)	0.48	-0.042	-0.028 (-0.742 to 0.685)	0.94

The implied direct effect is the total effect minus the indirect effect, with a delta-method standard error. For reflux disease the indirect effect exceeds the total effect, so the implied direct effect is positive; its confidence interval includes the null under every estimator because the total effect is itself imprecise, and it is therefore reported as hypothesis-generating rather than as evidence of a receptor-specific effect. Because the BMI to reflux disease MR-Egger intercept indicates directional pleiotropy (P = 1.6x10-5), the Egger-based indirect estimate is the pleiotropy-robust bound of the range. For BO/OAC the path-b estimate is supported by IVW only, so all estimates in those rows are exploratory. CI, confidence interval; IVW, inverse-variance weighted.

Table S20. Pre-registered secondary outcomes analysed post hoc in FinnGen R12.

Receptor	Instrument set	Outcome (FinnGen R12 endpoint)	Cases / controls	OR (95% CI)	P value	PP.H4	Region maximum -log10 P
GLP-1R	primary	Gastroduodenal ulcer (K11_GASTRODUOULC)	12,074 / 423,785	1.04 (0.73-1.46)	0.84	0.009	2.18
GLP-1R	correlated	Gastroduodenal ulcer (K11_GASTRODUOULC)	12,074 / 423,785	1.16 (0.94-1.43)	0.17	0.009	2.18
GIPR	primary	Gastroduodenal ulcer (K11_GASTRODUOULC)	12,074 / 423,785	1.46 (0.96-2.22)	0.077	0.032	2.69
GLP-1R	primary	Chronic gastritis (K11_CHRONGASTR)	12,441 / 423,785	0.87 (0.62-1.22)	0.41	0.011	2.67
GLP-1R	correlated	Chronic gastritis (K11_CHRONGASTR)	12,441 / 423,785	0.85 (0.69-1.05)	0.14	0.011	2.67
GIPR	primary	Chronic gastritis (K11_CHRONGASTR)	12,441 / 423,785	1.03 (0.68-1.56)	0.88	0.007	2.42
GLP-1R	primary	Gastric cancer (C3_STOMACH_EXALLC)	2296 / 378,749	1.47 (0.68-3.20)	0.33	0.027	3.00
GLP-1R	correlated	Gastric cancer (C3_STOMACH_EXALLC)	2296 / 378,749	1.33 (0.83-2.15)	0.24	0.027	3.00
GIPR	primary	Gastric cancer (C3_STOMACH_EXALLC)	2296 / 378,749	0.76 (0.29-1.94)	0.56	0.019	3.40
GLP-1R / GIPR	not applicable	Gastroparesis / upper-gastrointestinal dysmotility	Not available	Not analysable	-	-	-

These outcomes were registered as secondary or exploratory, had a priori power below 0.17 at the pre-analysis power assessment, and were not analysed for the original report; they were completed during revision and are reported irrespective of direction. The registered East Asian gastric cancer source (BioBank Japan) was replaced with a European FinnGen endpoint so that the outcome is ancestry-matched to the European cis-eQTL instruments. No estimate reached nominal significance. As for the oesophageal outcomes, the regional outcome signal was undetectable and colocalisation was uninformative. Gastroparesis and upper-gastrointestinal dysmotility could not be analysed because no adequately powered genome-wide association study exists; this was identified at the design stage. CI, confidence interval; OR, odds ratio.

Table S21. Illustrative clinical and epidemiological evidence used for triangulation.

Study (design)	Population	Outcome	Reported estimate	Direction	Interpretation for triangulation
Wang et al. 2024 (cohort)	T2D	Oesophageal cancer	HR 0.60 (0.42-0.86)	Protective	Directionally aligned
Ayoub et al. 2024 (propensity-matched cohort)	T2D	Oesophageal cancer, 7 y	0.04% vs 0.13%	Protective	Directionally aligned
Wu et al. 2025 (meta-analysis)	T2D	Oesophageal cancer	RR 0.46 (0.13-1.59)	Null/protective	Directionally aligned
Akerstrom et al. 2025 (cohort)	Nordic	Oesophageal cancer	No increased risk	Null	Directionally aligned
Dai et al. 2025 (cohort)	Obesity	Overall cancer	HR 0.83 (0.76-0.91)	Protective	Directionally aligned
Hsu et al. 2026 (cohort)	Obese, non-diabetic	Cancer (overall)	HR 0.59 (0.53-0.67)	Protective	Directionally aligned
Kuo et al. 2025 (cohort)	T2D	Gastrointestinal cancer	HR 0.29 vs insulin	Protective	Directionally aligned
RCT meta-analysis / SELECT trial	Trials	Malignancy	No excess	Null	Directionally aligned
Liu et al. 2024 (cohort)	T2D	reflux disease + complications	Short-acting HR 1.15; long-acting ~null	Harm (acute)	Divergent (timescale)
Niu et al. 2026 (PSM cohort)	T2D (*n*=230,415/group)	Oesophageal cancer	HR 0.74 (P=0.001)	Protective	Directionally aligned
Niu et al. 2026 (PSM cohort)	T2D (*n*=230,415/group)	Gastroparesis	HR 1.59 (P<0.001)	Harm (acute)	Not modelled (acute)
Liu et al. 2022 (pharmacovigilance)	FAERS	GI adverse events	Disproportionality signal	Harm (acute)	Not modelled (acute)
Ko et al. 2025 (dose-response meta-analysis)	BE patients	BE malignant progression (HGD/OAC)	OR 1.06 (1.02-1.10) per 5 kg/m²	Harm (adiposity)	Directionally aligned (BMI causal)
Zhu et al. 2024 (meta-analysis)	Post-bariatric surgery	Oesophageal cancer	OR 0.69 (0.51-0.95)	Protective	Directionally aligned (weight loss)
Xu et al. 2017 (meta-analysis)	T2D	Oesophageal adenocarcinoma	RR 1.43 (1.35-1.51)	Apparent harm	May reflect adiposity-related confounding

Estimates are on heterogeneous scales and are not directly comparable in magnitude; concordance is judged by direction and consistency with the mediated mechanism. Triangulation assesses consistency across designs, not proof; studies were selected illustratively for contextual interpretation rather than by systematic review.

Supplementary Figures

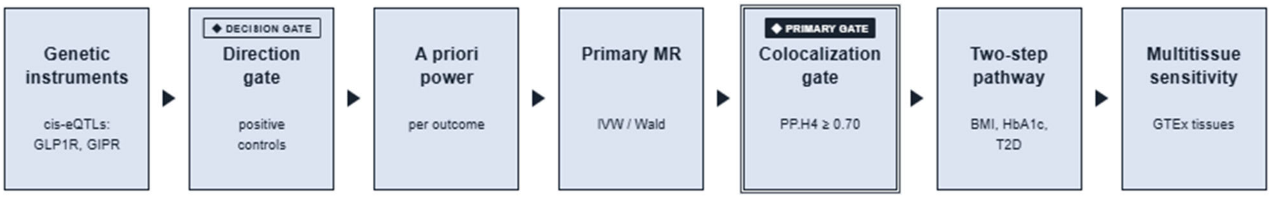

Figure S1. Analytic pipeline and prespecified decision gates.

The workflow proceeded from cis-eQTL instrument selection to positive-control validation, a priori power assessment, primary MR estimation (inverse-variance weighted/Wald ratio), colocalisation-based target-specific interpretation, exploratory two-step pathway analysis, and multitissue sensitivity analysis. Colocalisation (PP.H4 ≥0.70) was prespecified as the primary robustness criterion; the gate flagged the single-instrument OAC-only signal as non-interpretable.

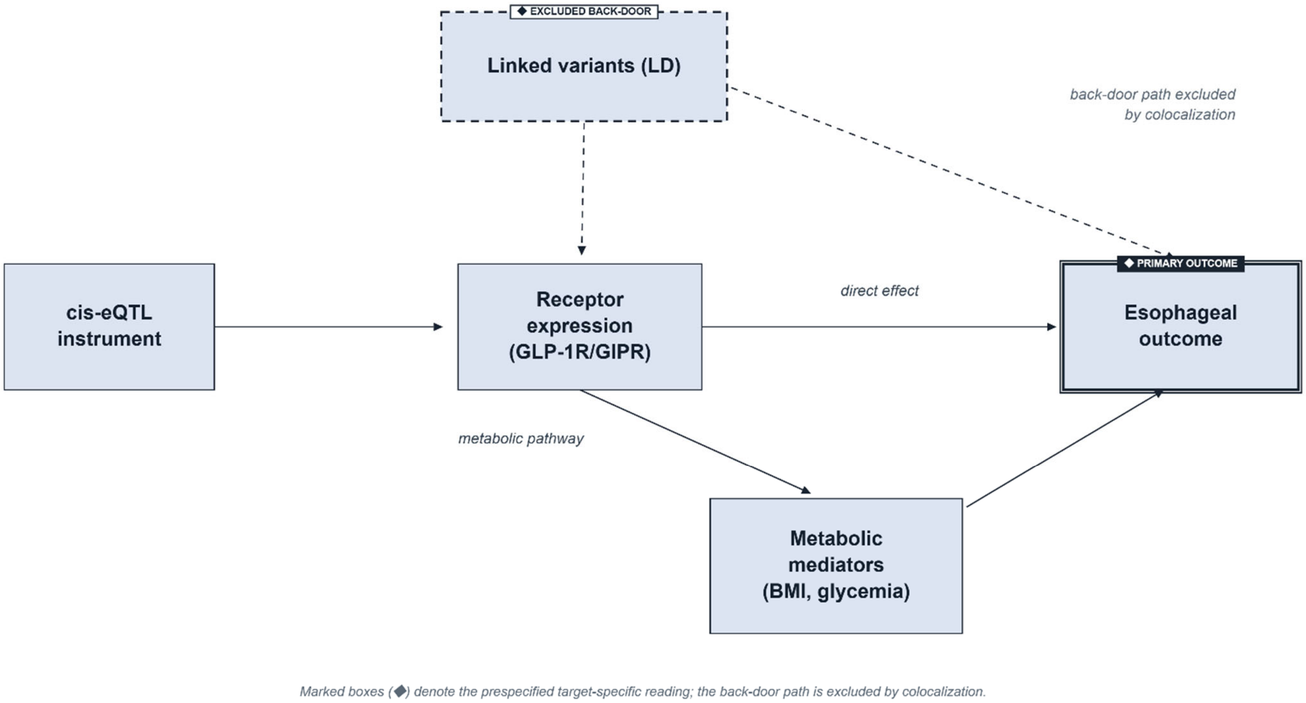

Figure S2. Assumed causal structure and identification assumptions (directed acyclic graph).

The cis-eQTL instrument proxies GLP-1R or GIPR target perturbation, which may relate to oesophageal outcomes through a receptor-specific path or through metabolic traits (body mass index, glycaemic traits). Linked variants can create non-target-specific associations; colocalisation was therefore required for target-specific interpretation.

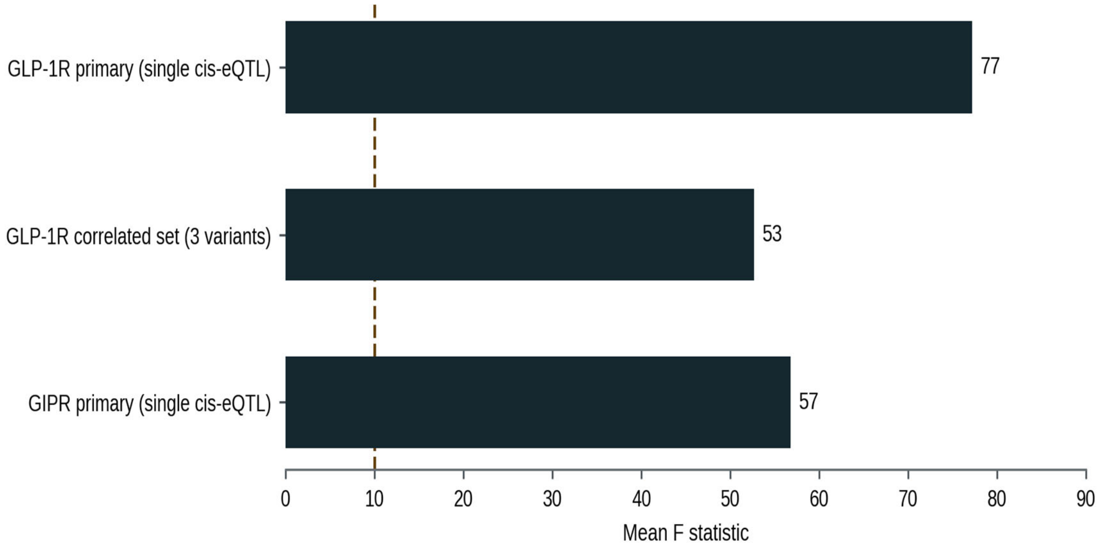

Figure S3. Instrument strength (mean F).

All instruments well above the weak-instrument threshold (F = 10).

Exposure	Outcome	β (95% CI)	P Value
**GLP-1R**	type 2 diabetes	-0.088 (-0.297 to 0.121)	P = .41
**GLP-1R**	BMI	-0.081 (-0.131 to -0.031)	P = .002
**GLP-1R**	HbA1c	-0.022 (-0.192 to 0.148)	P = .80
**GIPR**	HbA1c	-0.403 (-0.614 to -0.192)	P < .001

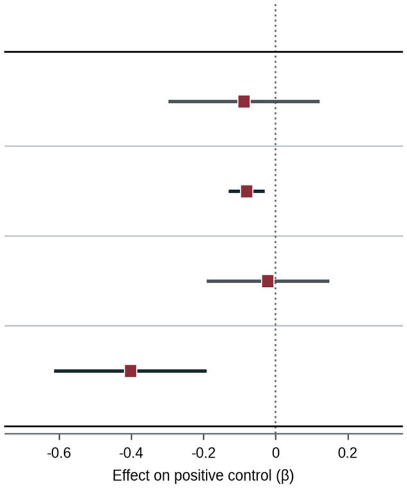

Figure S4. Positive-control direction gate.

Available positive-control estimates moved in the expected agonist-aligned direction; negative values indicate lower trait value or risk per genetically proxied higher receptor expression, consistent with the metabolic direction used for instrument validation. GIPR-BMI and GIPR-type 2 diabetes estimates were unavailable.

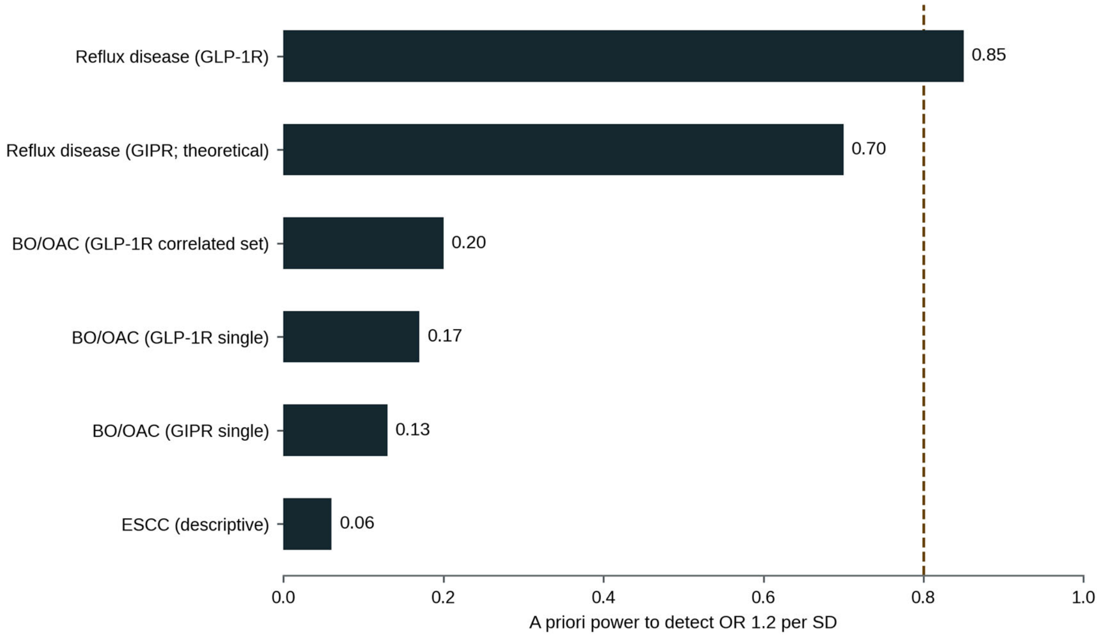

Figure S5. A priori power by outcome.

Power was adequate for the estimable GLP-1R-reflux disease analysis but limited for BO/OAC and descriptive/sensitivity outcomes. The GIPR-reflux disease value is a theoretical calculation conditional on instrument availability; no harmonised GIPR-reflux disease MR estimate was derived because the cis-eQTL instrument was unavailable in the reflux disease GWAS.

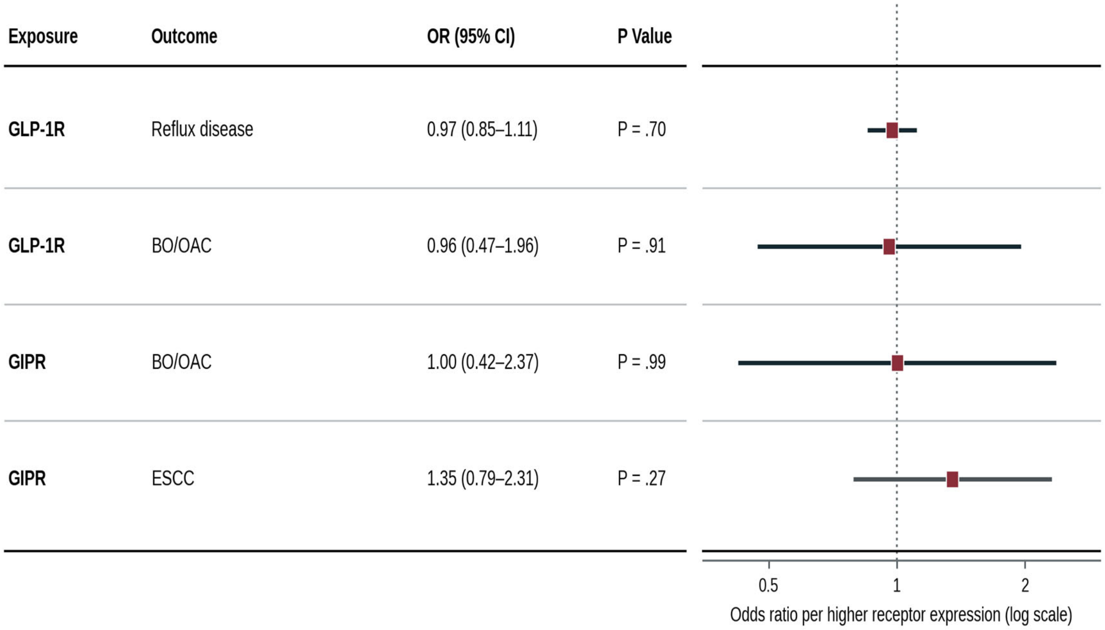

Figure S6. Summary of drug-target MR estimates by receptor-outcome pair. Odds ratios (95% CI) with P values are shown; no receptor-outcome pair reached the colocalisation threshold (all PP.H4 <0.70), but colocalisation was uninformative for these endpoints because the outcome loci carried no detectable association signal (Table S17).

Odds ratios are shown by colour and colocalisation posterior probabilities (PP.H4) by value; no pair reached PP.H4 0.70, and colocalisation was uninformative for these endpoints (Table S17).

Exposure	Outcome	β (95% CI)	P Value
**GLP-1R**	BO/OAC: receptor-outcome	-0.042 (-0.755 to 0.671)	P = .91
**GLP-1R**	BO/OAC: two-step (BMI)	-0.021 (-0.040 to -0.002)	P = .03
**GLP-1R**	Reflux disease: receptor-outcome	-0.026 (-0.159 to 0.107)	P = .70
**GLP-1R**	Reflux disease: two-step (BMI)	-0.061 (-0.100 to -0.023)	P = .002

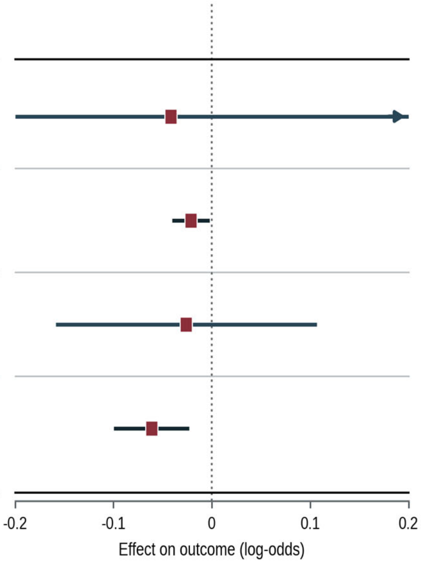

Figure S7. Exploratory GLP-1R-BMI two-step pathway estimate.

This decomposition supports Figure 3A and is hypothesis-generating; it does not represent mediation of a validated receptor effect.

Exposure	Outcome	β (95% CI)	P Value
BMI	Reflux disease	0.758 (0.708 to 0.808)	P < .001
BMI	BO/OAC	0.263 (0.093 to 0.433)	P = .002
HbA1c	Reflux disease	0.052 (-0.017 to 0.121)	P = .14
HbA1c	BO/OAC	0.060 (-0.083 to 0.203)	P = .41
Type 2 diabetes	Reflux disease	0.037 (0.011 to 0.063)	P = .006
Type 2 diabetes	BO/OAC	0.032 (-0.053 to 0.117)	P = .46

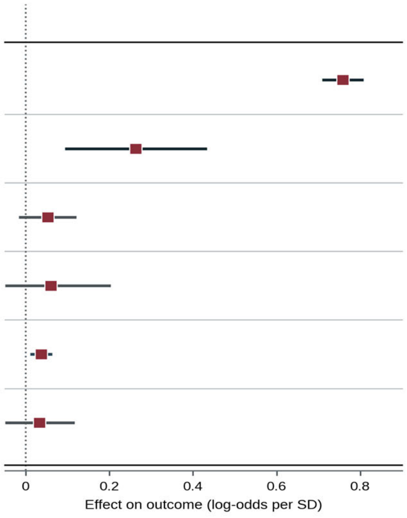

Figure S8. Metabolic trait-to-outcome MR estimates (path b).

BMI showed the most consistent association with reflux disease and BO/OAC in inverse-variance-weighted analyses (robust for reflux disease, IVW-only for BO/OAC); HbA1c and type 2 diabetes showed no consistent association with BO/OAC. These correspond to path b in the two-step pathway analysis.

Exposure	Outcome	OR (95% CI)	P Value
**GLP-1R**	BO/OAC (adenocarcinoma)	0.96 (0.47–1.96)	P = .91
**GIPR**	BO/OAC (adenocarcinoma)	1.00 (0.42–2.37)	P = .99
**GIPR**	ESCC (squamous comparator)	1.35 (0.79–2.31)	P = .27

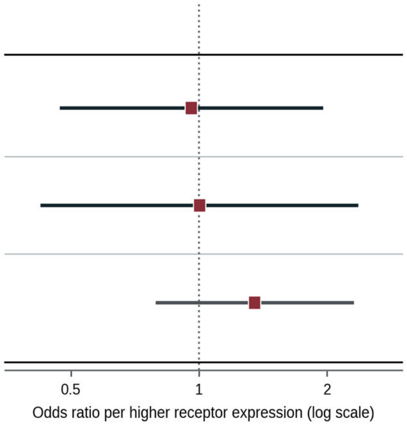

Figure S9. Descriptive comparison of the adenocarcinoma-axis outcome (BO/OAC) and the squamous-cell comparator (ESCC).

GIPR-ESCC was evaluated only as a descriptive comparator because of ancestry mismatch and limited power; no causal inference was drawn.

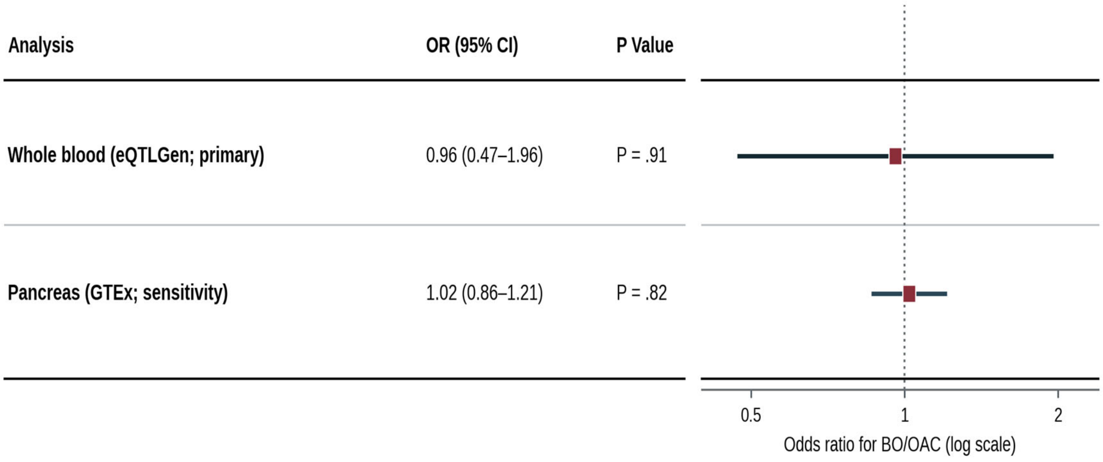

Figure S10. Tissue-specific GLP-1R cis-eQTL sensitivity analysis.

A pancreatic GLP-1R cis-eQTL yielded a BO/OAC estimate concordant with the primary blood eQTL-based analysis. No usable *GIPR* tissue cis-eQTL was detected in the queried tissues; this is a limited sensitivity analysis.

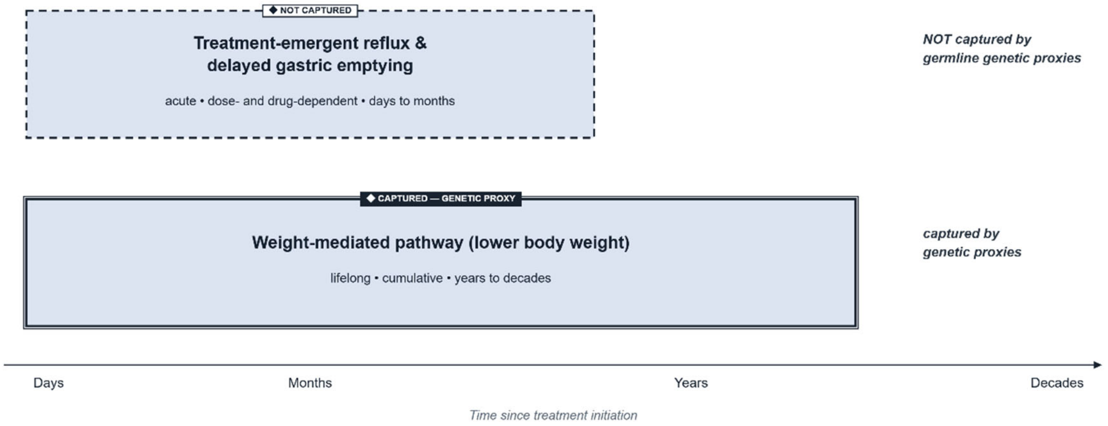

Figure S11. Distinct time scales for reflux-related symptoms and weight-mediated oesophageal risk modification.

Treatment-emergent reflux and delayed gastric emptying are acute, dose- and drug-dependent pathways not modelled by germline genetic proxies, whereas durable weight reduction acts over longer time scales and is more closely approximated by genetic analyses of adiposity.
